# Inhalable songorine-integrated lipid nanomedicine for targeted ARDS therapy *via* repairing endothelial barrier and inactivating NLRP3 inflammasome

**DOI:** 10.1016/j.apsb.2025.10.048

**Published:** 2025-11-04

**Authors:** Haiyan Wang, Zhi-Chao Sun, Chunlei Dai, Ran Liao, Ran Lin, Liying Wang, Wenjun Fu, Ruhe Zhang, Danwen Zheng, Zhongde Zhang, Jun Wu, Yuntao Liu

**Affiliations:** a State Key Laboratory of Traditional Chinese Medicine Syndrome, Guangzhou University of Chinese Medicine, Guangzhou 510006, China; b Chinese Medicine Guangdong Laboratory, Guangzhou University of Chinese Medicine, Zhuhai 519031, China; c Bioscience and Biomedical Engineering Thrust, Systems Hub, The Hong Kong University of Science and Technology (Guangzhou), Guangzhou 511400, China; d Division of Life Science, The Hong Kong University of Science and Technology, 999077, Hong Kong, China; e Department of Hematologic Oncology, Sun Yat-sen University Cancer Center, State Key Laboratory of Oncology in South China, Collaborative Innovation Center for Cancer Medicine, Guangdong Provincial Clinical Research Center for Cancer, Guangzhou 510060, China

**Keywords:** Acute respiratory distress syndrome, Songorine, Liposome, Lung-targeted property, Anti-inflammation, Anti-oxidation, Endothelial barrier repair, Inactivation of NLRP3 inflammasome

## Abstract

Acute respiratory distress syndrome (ARDS) is a life-threatening disease. In the clinical management of ARDS, current treatments such as glucocorticoids and protease inhibitors encounter significant challenges due to their high toxicity, limited administration routes, or poor targeting. These limitations highlight the urgent need for innovative therapeutic strategies. Songorine (Son), a compound derived from the herb *Aconitum carmichaelii* Debeaux, possesses good antioxidant and anti-inflammatory properties, exhibiting great potential for treating ARDS. However, its clinical application is partially constrained by low aqueous solubility and uncertain efficacy for ARDS. In this study, we developed a lung-targeted lipid nanomedicine by encapsulating Son in dipalmitoyl phosphatidylcholine (DPPC) liposomes (Son@liposome, Son-lipo). In a lipopolysaccharide-induced ARDS mouse model, we demonstrated that Son-lipo effectively targeted inflamed lung tissues with commendable biocompatibility. Further, Son-lipo significantly alleviated multiple ARDS phenotypes such as endothelial barrier damage, lung edema, pulmonary dysfunction, and alveolar lesion, which involved uncontrolled inflammation, oxidative stress, and cell apoptosis. RNA sequencing and Western blotting analyses revealed that Son-lipo inhibited the activation of the TLR4/NF-*κ*B/NLRP3 pathway responsible for ARDS. In conclusion, our study successfully developed an inhalable lipid-nanomedicine (Son-lipo) as a novel therapeutic strategy for ARDS. It elucidates the formulation's ability to mitigate ARDS by repairing the endothelial barrier and reversing the inflammatory microenvironment, thereby providing a promising candidate drug for improving clinical management of ARDS.

## Graphical abstract

In this study, an inhalable lipid-nanomedicine (Son-lipo) was successfully developed, and it was found to alleviate ARDS by repairing the endothelial barrier and reversing the inflammatory microenvironment.

**Figure:**
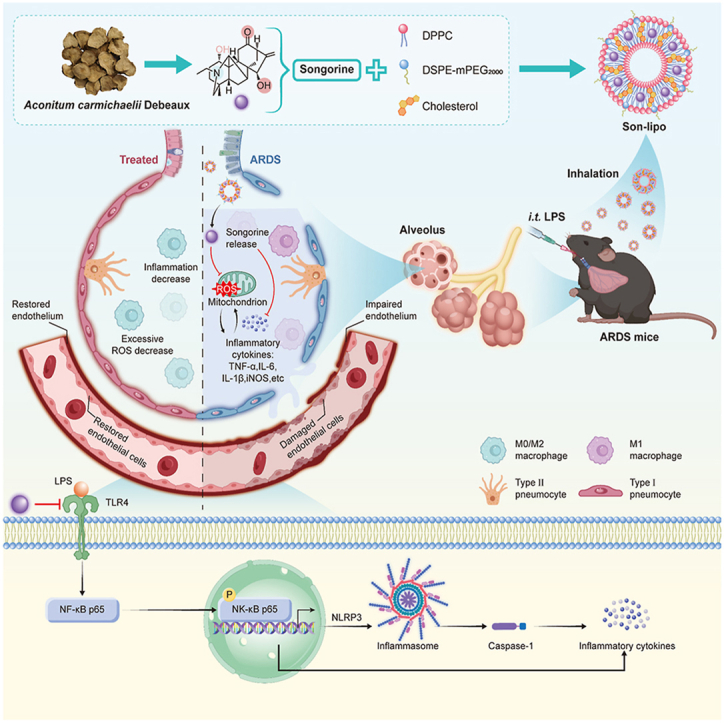

## 1 Introduction

Acute respiratory distress syndrome (ARDS) is a critical condition in respiratory medicine, characterized by increased vascular permeability and severe pulmonary inflammation. It is recognized as one of the highest-mortality complications linked to a wide range of diseases^1^, ^2^, ^3^, ^4^. Therefore, ARDS remains a considerable challenge. At present, among the principal clinical interventions employed for the management of ARDS, mechanical injury caused by mechanical ventilation, nephrotoxicity of inhaled vasodilators, and strong immunosuppressive effects of potent anti-inflammatory glucocorticoids have been shown to impede the subsequent treatment and rehabilitation of patients^5^, ^6^, ^7^. Consequently, it is urgent to develop safe, efficacious, and novel therapeutic agents for ARDS.

Anti-inflammatory compounds derived from herbs show considerable therapeutic potential in treating ARDS. Songorine (Son), a natural diterpenoid alkaloid extracted from *Aconitum carmichaelii* Debeaux, is known for its strong antioxidant and anti-inflammatory properties^8^. Nonetheless, the activities of Son in ARDS therapy remain ambiguous. Poor solubility and lack of targeted delivery significantly limit its application in ARDS treatment. Thus, selecting suitable carriers to improve water solubility and ensure targeted delivery to inflamed lung tissues is crucial for the clinical translation of Son.

Compared to conventional drug delivery methods, nano-based systems offer significant potential in treating ARDS due to their advantages of improved water solubility, targeted delivery capabilities, and reduced side effects^9^, ^10^, ^11^, ^12^. However, limited drug-loading capacity, insufficient stability, and complex preparation processes are key factors that hinder the application of some nanomedicines^13^, ^14^, ^15^. Thus, selecting appropriate nanocarriers is crucial for advancing nanomedicines and facilitating their clinical translation. Liposomes (Lipo), a type of FDA-approved nanocarrier, have been successfully used in developing a wide range of clinical pharmaceuticals^16^, ^17^, ^18^, ^19^, ^20^. In addition, Lipo can be used for targeted delivery, structural modulation, and controlled drug release through component adjustment or optimization of preparation methods^21^, ^22^, ^23^, ^24^. Therefore, liposomes are increasingly being utilized to deliver insoluble natural compounds for the treatment of ARDS^25^, ^26^, ^27^.

Disruption of the vascular endothelial barrier is a key characteristic in the pathogenesis of ARDS^28^^,^^29^. Restoring this barrier damage is essential for the prevention and treatment of ARDS^30^, ^31^, ^32^, ^33^. The compromised pulmonary endothelial barrier results in heightened pulmonary vascular permeability and infiltration of inflammatory cells, which promotes the propagation of inflammation and exacerbates ARDS^34^. In particular, activated neutrophils excessively generate reactive oxygen species (ROS) during oxidative stress, further aggravating the disruption of tight junctions between endothelial cells and accelerating the progression of ARDS^35^^,^^36^. Thus, an antioxidant combined with anti-inflammatory therapy may serve as a potentially effective intervention to repair the compromised endothelial barrier in ARDS.

Nebulized inhalation is a drug administration strategy that utilizes nebulizer devices to aerosolize liquid medication into aerosol particles with diameters ranging from 0.01 to 10 μm. These aerosolized particles are then inhaled, allowing them to enter the airways and deposit in the lungs, thereby exerting therapeutic effects for the prevention and treatment of pulmonary diseases^37^^,^^38^. Nebulized inhalation of liposomal formulation enables efficient localized pulmonary drug delivery, thereby enhancing the drug's therapeutic efficacy. In detail, it can increase drug bioavailability, reduce systemic exposure, and diminish toxicity^39^. Therefore, nebulized inhalation was selected as the administration route to minimize adverse reactions in mice.

**Scheme 1:**
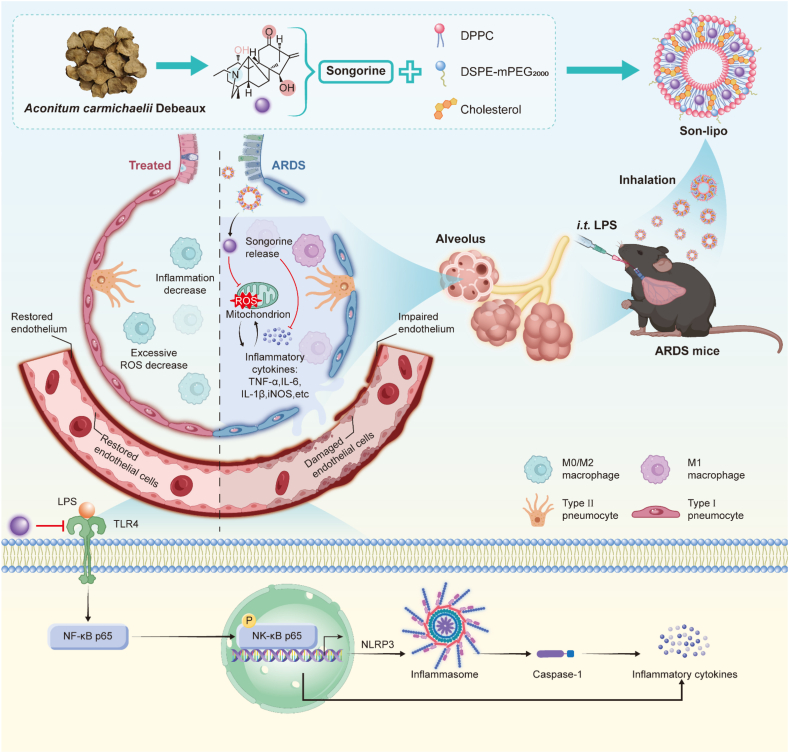
The synthesis of Son-lipo for ARDS therapy. Son-lipo was prepared by intermingling Son in a DPPC Lipo. Then, Son-lipo exhibited anti-inflammatory effects and facilitated the repair of the vascular endothelial barrier *via* inhibiting the activation of the NLRP3 inflammasome.

In this study, we developed a lung-targeted Son-loaded liposome (Son-lipo) composed of dipalmitoyl phosphatidylcholine (DPPC), DSPE-mPEG_2000,_ cholesterol, and Son for ARDS treatment (Scheme 1). DPPC, as a pulmonary surfactant, provides lung-targeting capability for Lipo^40^. DSPE-mPEG_2000_ contributes to prolonged systemic circulation time and enhanced drug encapsulation and loading efficiency^41^. Cholesterol, an auxiliary component of liposomes, aids in extending the therapeutic efficacy and circulation time of the formulation^42^. Both *in vitro* and *in vivo* experiments demonstrated that Son-lipo exhibited commendable stability, lung-targeting capability, and reliable biocompatibility. Importantly, inhalable Son-lipo was confirmed to repair the endothelial barrier and improve lung function through anti-oxidative stress, anti-inflammation, and anti-apoptosis, thereby ameliorating ARDS in mice. Mechanically, Son-lipo inhibited the activation of the TLR4/NF-*κ*B/NLRP3 pathway responsible for ARDS. Taken together, this study for the first time elucidates that the Son-lipo alleviates ARDS by repairing the endothelial barrier and inactivating NLRP3 inflammasome, hence providing a promising strategy for the clinical treatment of ARDS.

## 2 Materials and methods

### 2.1 Cell culture

The RAW264.7 and human umbilical vein endothelial cells (HUVEC) were gifted by Professor Jun Wu from the Hong Kong University of Science and Technology (Guangzhou). RAW264.7 and HUVEC cells were cultured in DMEM containing 10% FBS (ExCell Bio, FSD500, Suzhou, China) and 1% penicillin-streptomycin solution (Solarbio, P1400, Beijing, China) at 37 °C with 5% CO_2_ addition.

### 2.2 Reagents

Son was obtained from Chengdu Puruifa Technology Development Co., Ltd. (509-24-0, Chengdu, China). High-glucose DMEM (containing 4.5 g/L d-glucose, l-glutamine, and 110 mg/L sodium pyruvate) was from GIBCO (C11995500BT, NY, USA). DPPC was from Aladdin Biochem Technology Co., Ltd. (D130424, Shanghai, China). DSPE-mPEG_2000_ was from Hunan Huateng Pharmaceutical Co., Ltd. (MF001096-2K, Hunan, China). Cholesterol was from Macklin Biochemical Technology Co., Ltd. (C6213, Shanghai, China). Dil was from·Yeasen·Biotechnology·Co., Ltd. (40726ES10, Shanghai, China). Dir fluorescent probe was from MeilunBio (MB12482, Dalian, China). Lipopolysaccharide (LPS) was from·Sigma-Aldrich (L2880, Darmstadt, Germany). Chloroform was from Sinopharm Chemical Reagent Co., Ltd. (67-66-3, Shanghai, China)

### 2.3 Preparation of Lipo and Son-lipo

Lipo and Son-lipo were prepared using the thin-film hydration method. Specifically, 44.25 mg DPPC, 5.80 mg cholesterol, 2.00 mg DSPE-mPEG_2000_, 5 mL chloroform, and 10.00 mg Son were added to a 500 mL round-bottomed flask and dissolved thoroughly. A rotary evaporator (Buchi, R-300, Switzerland) was used to remove solvent and form a lipid film at 230 mbar. The film was then hydrated with 5 mL phosphate buffer solution (PBS) on magnetic stirrer at 60 °C, 300 rpm for 30 min (DLAB, MS-H-ProA, Beijing, China). The re-suspended lipid film was sonicated using an ultrasonicator (SCIENTZ, SCIENTZ-IID, Ningbo, China) on ice for about 30 min to obtain dispersed multilamellar liposomes. The suspension was filtered through a 0.22 μm microporous membrane (Biosharp, BS-PES-22-S, Hefei, China) after ultrasonic dispersion, collected, and transferred to an ultrafiltration tube (Merck Millipore®, UFC9030, Darmstadt, Germany). It was then centrifuged at 4 °C, 3000 rpm for 1 h (Eppendorf, 5420R, Hamburg, Germany). Finally, PBS was used to adjust the volume of Son-lipo in the ultrafiltration tube to 3 mL. To prepare liposomes, 6.00 mg of Son was omitted, and the other procedures remained the same as mentioned above. For fluorescence visualization, fluorescent dyes (Dir and Dil) were added to label Lipo or Son-lipo during the lipid film preparation process.

### 2.4 Characterization of Lipo and Son-lipo

Initially, freshly prepared Lipo and Son-lipo were diluted in ultrapure water at a ratio of 1:10 and then sonicated for 30 s. Their physiological parameters, including size distribution, polydispersity index (PDI), and zeta potential, were then analyzed using a particle size analyzer (Brookhaven, NanoBrook 90 Plus PALS, NY, USA). For morphological examination, a 2.5 mmol/L solution of the newly prepared Lipo and Son-lipo was applied onto a copper grid and allowed to dry in a biochemical incubator at 37 °C for 48 h. The morphology of the samples was observed and imaged using a transmission electron microscope (TEM; JEOL Ltd, JEM-2100F, Tokyo, Japan).

### 2.5 Cell counting kit-8 (CCK-8) assays

The cytotoxicity of the pharmaceuticals was assessed using the CCK-8 kit (GLPBIO, GK10001-5, CA, USA). RAW264.7 and HUVEC cells were seeded into 96-well culture plates at densities of 1 × 10^4^ and 5 × 10^3^ cells/well, respectively. Various concentrations of Lipo and Son-lipo were applied for 24 h. Subsequently, 100 μL of CCK-8 working solution was added to each sample and incubated at 37 °C for 2 h. The absorbance of each well was ultimately measured at 450 nm with a microplate reader (BioTek, SYNERGY H1, VT, USA).

### 2.6 Live/dead staining

Live/dead staining was performed according to the protocol of the Calcein/PI Cytotoxicity Assay Kit (Beyotime, C2015S, Shanghai, China). HUVEC cells were seeded into 6-well culture plates (Corning, CLS3516, NY, USA). When the cells reached 60%‒70% confluence, they were treated with 250 μmol/L Son-lipo, 250 μmol/L Son, and an equivalent volume of Lipo, respectively. After 12 or 24 h, the cells were rinsed with PBS, stained with Calcein AM to identify live cells and PI to identify dead cells, and then incubated at 37 °C for 30 min. The cells were washed again and subsequently immersed in fresh DMEM. Images of the stained cells were captured at 100× magnification using an inverted fluorescence microscope (Nikon, ECLIPSE Ti2-E, Tokyo, Japan).

### 2.7 Cytoskeleton staining

Cytoskeleton staining was conducted according to the instructions of the FITC Phalloidin Staining Kit (Yeasen, 40735ES75, Shanghai, China). HUVEC cells were seeded in 0.17 mm glass-bottomed Petri dishes (Biosharp, BS-15-GJM, Hefei, China) and allowed to grow to 60%‒70% confluence. Then, the cells were treated with 250 μmol/L Son-lipo, 250 μmol/L Son, and Lipo (equal volume to that of Son-lipo), respectively. After 12 or 24 h, the cells were washed with PBS and fixed with 4% paraformaldehyde for 10 min. Subsequently, the cells were permeabilized with 0.5% Triton X-100 and then treated with 100 nmol/L FITC phalloidin (diluted with 1% bovine serum albumin) for 30 min at room temperature in the dark. The cells were imaged using a 60× oil immersion lens on a laser scanning confocal microscope (Zeiss, LSM710, Oberkochen, Germany).

### 2.8 Hemolysis ratio test

Initially, 2 mL of fresh blood was extracted from the ocular cavities of rats and mixed with 10 mL of PBS containing 1 × 10^4^ units of sodium heparin (Solarbio, 9041-08-1, Beijing, China). The diluted blood was centrifuged at 3000 rpm for 10 min to isolate the erythrocytes (Eppendorf), washed until the supernatant was clear, and then diluted with PBS to achieve a 2% cell suspension. The diluted erythrocytes and varying doses of Son or Son-lipo were added to 1.5 mL centrifuge tubes and incubated at RT for 3 h. Diluted erythrocytes were directly interacted with ultrapure water, and PBS served as the positive and negative controls, respectively. Finally, all tubes were centrifuged at 1000 rpm for 15 min to isolate the supernatant (Eppendorf), which was then measured at 540 nm using a microplate reader (BioTek). The hemolysis rate was determined using Eq. (1):

(1) Hemolysis rate (%) = (Ap−Ab)/(At−Ab) × 100%

where Ap represents the drug absorbance value, At denotes the positive control, and Ab signifies the negative control.

### 2.9 Cellular uptake

RAW264.7, MH-S, and HUVEC cells were seeded in 6-well plates and incubated overnight. Dil or Dil@Son-lipo was incubated with cells for 1, 4, 8, or 12 h. After the cells were washed and fixed, the nuclei were stained with Hoechst33342 (Thermo Fisher Scientific, H3570, MA, USA). Finally, samples were photographed and observed by a 40× oil immersion lens on a confocal macroscope (Zeiss).

### 2.10 Flow cytometry

Firstly, HUVEC cells were seeded into a 6-well culture plate at a density of 2 × 10^5^ cells per well and cultivated until 40%‒50% confluence. Subsequently, 250 μmol/L of Son, 250 μmol/L of Son-lipo, and Lipo (equal volume to that of Son-lipo) were added, and co-incubated with 2 μg/mL of LPS for 24 h. Following incubation, the harvested cells and supernatant were treated with 0.02% EDTA-free trypsin, and staining was conducted according to the protocol of the Annexin V, FITC Apoptosis Detection Kit (DOJINDO, AD10, Shanghai, China). Finally, the apoptotic ratio of the HUVEC cells was evaluated using a flow cytometer (Agilent, ACEA NovoCyte, CA, USA). The resultant data were analyzed using FlowJo software (BD, v10.0.8, OR, USA).

### 2.11 Intracellular ROS detection

The Reactive Oxygen Species Assay Kit (Beyotime, S0033S, Shanghai, China) was used. HUVEC cells were seeded in a 6-well plate and cultured until they reached 60%‒70% confluence. The medium was then replaced with freshly prepared DMEM containing 250 μmol/L Son, 250 μmol/L Son-lipo, or Lipo (equal volume to that of Son-lipo), supplemented with 1 μg/mL LPS, and cells were incubated for 24 h. Subsequently, the cells were incubated with DCFH-DA (Beyotime) at a final concentration of 10 μmol/L at 37 °C for 20 min. Following incubation, the cells were washed with PBS. The fluorescence intensity of the DCFH-DA probe was then evaluated through flow cytometry, and the data were then analyzed by Image J and FlowJo v10.0.8 software (BD).

### 2.12 Cell scratch assay

HUVEC cells were seeded at a density of 1 × 10^5^ cells per well in six-well plates. When cell confluence reached 100%, a scratch wound was created using a sterile 200 μL pipette tip. Then, the cells were gently washed three times with PBS. The Control group received complete medium only; the Model group was treated with complete medium containing 500 ng/mL LPS; the Son, Lipo, and Son-lipo groups were co-incubated with 500 ng/mL LPS and 250 μmol/L of the respective drug formulations. Images of the wound area were captured at 0 and 24 h.

### 2.13 Animal care and use

The C57BL/6 mice (6‒8 weeks, male) were provided by Guangdong Vital River Technology Co., Ltd. (Guangzhou, China). All experimental procedures were executed according to the protocols approved by Experimental Animal Research Center of the Second Affiliated Hospital of Guangzhou University of Chinese Medicine (Permit No. 2023017).

### 2.14 ARDS animal model and treatments

Mice were randomly divided into five groups: Control, Model, Lipo, Son, and Son-lipo. Mice in the Model, Lipo, Son, and Son-lipo received 5 mg/kg of LPS *via* intratracheal (i.t.) injection on Day 0, while the Control was administered with an equal volume of PBS instead of LPS. After that, 6 mL of 250 μmol/L Son, 250 μmol/L Son-lipo, or lipo (equal volume to Son-lipo) were inhaled by mice for 20 min twice a day. On Day 3, the mice were weighed, subjected to lung function tests, and then sacrificed. Lungs, livers, spleens, hearts, kidneys, and blood were harvested for further analysis. Throughout this process, the body weight of the mice was recorded daily. Additionally, some mice were euthanized 24 h after LPS stimulation for bronchoalveolar lavage fluid (BALF) collection.

### 2.15 Septic animal model and treatments

Mice were randomly assigned to six groups: Control, Model, Lipo, Son, Son-lipo, and Dexamethasone (Dex). The Model, Lipo, Son, Son-lipo, and Dex groups were intraperitoneally injected (i.p.) with 20 mg/kg LPS on Day 0, whereas the Control group received an equivalent volume of PBS. Nebulized inhalation of 6 mL Son (250 μmol/L), Son-lipo (250 μmol/L), Lipo (volume equivalent to Son-lipo), or Dex (5 mg/kg) was administered for 20 min. After 24 h, mice were weighed, and the lung, liver, spleen, heart, kidney, and blood samples were collected for subsequent analyses.

### 2.16 *In vivo* biodistribution

Dir (red fluorescent dye) was applied to label Son-lipo (Dir@Son-lipo; mass ratio of Dir and Son-lipo, 1:50) using the same fabrication process as that of Son-lipo. ARDS animal model was first established in mice *via* i.t. 5 mg/kg LPS. Following 4 h of LPS exposure, the mice received intraperitoneal injections of either free Dir or Dir@Son-lipo at a volume equivalent to that of Son-lipo. At 1, 12, and 24 h post-injection, the mice were euthanized, and their lungs, livers, hearts, spleens, and kidneys were harvested for imaging using a living imaging system (IVIS) (BLT, AniView Kirin, Guangzhou, China). The acquired data were subsequently analyzed based on fluorescence intensity.

### 2.17 Pharmacokinetic studies

Mice were acclimated for 7 days, and then randomly and equally assigned to Son and Son-lipo groups. After fasting with free access to water for 12 h, 12 mL of 250 μmol/L Son or 250 μmol/L Son-lipo was administered by nebulized inhalation for 40 min. Blood samples (0.3 mL) were collected from the orbital venous plexus at 5, 15, 30 min, 1, 2, 4, 8, and 24 h after drug administration into EDTA tubes and centrifuged at 3500 rpm for 20 min to separate plasma, which was subsequently stored at −80 °C. Additionally, lung tissues were excised at 5, 30 min, and 8 h post-administration, and then washed, weighed, and homogenized in PBS. Drug concentrations were quantified by LC–MS/MS (SCIEX, QTRAP 6500+, Shanghai, China). Pharmacokinetic parameters were calculated using Phoenix WinNonlin software (Certara, version 8.1, NJ, USA).

### 2.18 Lung function and lung index

Lung function indices were assessed using noninvasive whole-body volumetric tracing (EMMS, WBP, London, UK) in ARDS mice prior to euthanasia. Respiratory data were collected from mice for 10 min in a quiet and dark environment in a lit plethysmography chamber. Four indices were examined within the same respiratory frequency range to assess lung function: expiratory flow at 50% lung volume (EF50), enhanced pause (Penh), end-expiratory pause (EEP), as well as tidal volume (TV) normalized by body weight.

EF50 serves as an indicator for assessing airway resistance and airflow limitation. A decrease in EF50 typically reflects airway narrowing or obstruction. Penh represents changes in expiratory resistance and respiratory rhythm, indirectly reflecting airway resistance and lung compliance. TV/Body denotes the ratio of tidal volume (the volume of air inhaled or exhaled per breath) to body weight. Normalizing tidal volume to body weight allows for more accurate comparisons of pulmonary ventilation function across animals with different body masses, minimizing the confounding effects of weight differences. EEP reflects the residual alveolar pressure that helps prevent alveolar collapse and maintain alveolar patency. Modulation of EEP is crucial for improving gas exchange and overall lung function.

The complete fresh lung tissues from each mouse were harvested and weighed (*W*_1_). The relative murine body weight was recorded at the time of sacrifice (*W*_2_). The lung index was as shown in Eq. (2):

(2) Lung index (%) = *W*_1_/*W*_2_ × 100%

### 2.19 BALF collection and analysis

Following anesthesia, mice underwent intubation *via* a small neck incision, followed by perfusing the lung with 0.7 mL of pre-cooled sterile physiological saline three times. BALF was collected and centrifuged at 4 °C at 3000 rpm for 10 min (Eppendorf). The supernatant was used to measure the total protein concentration by employing the BCA assay kit (Beyotime, P0010S, Shanghai, China). The cells were lysed using RBC lysate on ice for 15 min, vortexed intermittently, then centrifuged at 450×*g* for 10 min at 4 °C. The freshly produced cell spheres were resuspended in 3 mL of PBS, and 20 μL of the cell suspension was used for counting with a cell counter (Countstar, IC 1000, Shanghai, China).

### 2.20 Histopathological evaluation

Fresh lung tissues were fixed in a 4% paraformaldehyde solution for 24 h, then embedded in paraffin and sectioned to a thickness of 4 μm. Slides were prepared by drying them to remove moisture and stained with hematoxylin and eosin (HE; Yeasen, 60524ES60, Shanghai, China) according to standard procedures. The stained slides were then dried and sealed. Lung tissues were analyzed using the Pannoramic MIDI (3DHISTECH, Budapest, Hungary) and observed with a software (3DHISTECH, CaseViewer 2.4.0, Budapest, Hungary). The other tissues’ sections were imaged using a 100 × magnification of an optical microscope (Olympus, BX53+DP72, Tokyo, Japan).

### 2.21 Immunohistochemistry assay

Lung tissues were routinely deparaffinized and dehydrated after paraffin sectioning and rinsed in PBS. Sections were heated in citrate buffer (pH 6.0) on high heat for 15 min, maintained on low heat for 10 min, cooled naturally for about 120 min, and rinsed in PBS. The 3% H_2_O_2_ was incubated at RT for 15 min and rinsed in PBS. Normal goat serum (Sigma, G6767, MO, USA) was used to block the samples for 1 h at 37 °C. The primary antibody ZO-1 (1:1500, Proteintech, 21773-1-AP, Wuhan, China), Claudin-5 (1:700, Servicebio, GB111290, Wuhan, China), NLRP3 (1:200, Adipogen, AG-20B-0014, CA, USA), pro-caspase-1 and caspase-1 (1:500, Adipogen, AG-20B-0042, CA, USA) were added and incubated with the samples at 4 °C overnight. The next day, the samples were incubated with the secondary antibodies at room temperature for 2 h. Then, Diaminobenzidine (DAB; Beyotime, P0203, Shanghai, China) treatment was carried out for 4‒5 min and terminated by rinsing with distilled water. Hematoxylin (Beyotime, C0105, Shanghai, China) was used to counterstain nuclei for 5 min. Afterwards, the sections were rinsed with tap water for 5 min, dehydrated in a gradient of alcohol, and then sealed with neutral gum and observed under a 100× magnification of an optical microscope (Olympus).

### 2.22 Subcellular localization and immunofluorescence

HUVEC cells were seeded in 0.17 mm glass-bottomed petri dishes (Biosharp) and cultured until 100% confluence was achieved. Cells were subsequently exposed to 1 μg/mL LPS. In the treatment groups, 250 μmol/L Son and 250 μmol/L Son-lipo were added to the corresponding dishes. The cells were fixed with 4% paraformaldehyde for 10 min, permeabilized with 0.5% Triton X-100 for 10 min, and finally blocked with 5% BSA for 1 h. Subsequently, the cells were incubated with tight junction factor ZO-1 antibody (1:1000, Proteintech, 21773-1-AP, Wuhan, China) overnight. The following day, cells were treated with donkey anti-rabbit Cy3 antibody (1:200, Biolegend, 406402, CA, USA) for 1 h, and nuclei were stained with Hoechst 33342 (Thermo Fisher Scientific). Samples were imaged using a 40× oil immersion lens on a laser scanning confocal microscope (Zeiss).

Immunofluorescence staining was conducted on 4 μm-thickness paraffin slices of lung tissue. Dehydration and antigen retrieval of the sections were performed prior to staining. The staining method was as described above. The primary antibody was the endothelial barrier marker VE-Cadherin (1:100, Santa Cruz, sc-9989, CA, USA). The secondary antibody was goat anti-mouse Cy3 (1:200, Biolegend, 405309, CA, USA). Finally, the sections were sealed and imaged using a 40 × oil immersion lens of a confocal microscope (Zeiss).

### 2.23 Biosafety evaluation

Twenty C57BL/6 mice were divided into four groups: Control, Lipo (equal volume to that of Son-lipo), Son (250 μmol/L), and Son-lipo (250 μmol/L). The Control group received a PBS administration. After injection, the general physiological conditions, signs of intoxication, and mortality of mice were observed on Days 1, 2, and 3. The animals’ body weight was recorded every day. Blood biochemical and blood routine examinations were performed, and the heart, liver, spleen, lung, and kidney were taken for HE staining to observe the pathological conditions.

### 2.24 RNA sequencing (RNA-seq) and data analysis

Total RNA of lung tissues was extracted by using a Universal RNA Extraction CZ kit (ONREW, RNC643, Foshan, China). The purity, integrity, and quantity of RNA were assessed using denaturing agarose gel electrophoresis. Subsequently, RNA was reverse-transcribed to establish the cDNA libraries following the protocol of VVAHTS® Universal V8 RNA-seq Library Prep Kit for Illumina (Vazyme, NR605-0, Nanjing, China). Sequencing of the cDNA libraries was conducted for subsequent bioinformatics analysis using the platform (Illumina, NovaSeq 6000, CA, USA).

The raw data were checked *via* FastQC (Babraham Institute, v0.11.2, Cambridge, UK) and then processed with Skewer (ICT, v0.2.2, Beijing, China). Paired-end reads were generated after removing low-quality reads. The clean data were aligned to the NM_10 mouse reference genome by using STAR (Cold Spring Harbor Laboratory, 2.7.11b, NY, USA). The mRNA expression data were generated by StringTie (NHGRI, v1.3.1c, MD, USA), and different gene expression was analyzed *via* DESeq2 (EMBL, v1.16.1, Heidelberg, Germany). Differentially expressed genes (DEGs) were identified with criteria of *P* < 0.05 and fold change ≥2. Finally, the GO and KEGG enrichment analysis of the DEGs was conducted through topGO and the KEGG database, respectively. The volcano plots, bubble diagrams, and other figures were graphed.

### 2.25 Quantitative reverse transcription PCR (RT-qPCR)

To assess the anti-inflammatory effect of the drugs, RAW264.7 cells were seeded in 6-well plates to grow to 60%‒70% confluence. They were then incubated with 250 μmol/L Son or 250 μmol/L Son-lipo diluted in 2 mL of fresh serum-free medium for 10‒12 h. The cells were then stimulated with 500 ng/mL LPS diluted in 2 mL of serum-free medium for 4 h. Afterwards, the cells were incubated again with Son or Son-lipo as described above for 6 h. Finally, the cells were collected for RT-qPCR analysis.

Total RNA was extracted from RAW264.7 cells, HUVEC cells, and lung tissues using RNA Simple Total RNA Kit (Tiangen Biotech, DP419, Beijing, China). PrimeScript RT reagent Kit with gDNA Eraser Perfect Real Time (Takara Bio, RR047A, Beijing, China) was used to convert the RNA samples into cDNA. The cDNA templates were subsequently subjected to RT-qPCR using SYBR Green qPCR Mix (Accurate Biology, AG11701, Hunan, China) on an ABI ViiA™ 7 system (Thermo Fisher Scientific, CA, USA). The housekeeping gene, *α*-Tubulin, was used as an endogenous control. The relative normalized mRNA expression of genes was calculated according to the 2^−ΔΔCT^ method. The specific primers were listed in Supporting Information Table S1.

### 2.26 Western blot analysis

Total protein was extracted from lung tissues or HUVEC cells using RIPA buffer (Beyotime, P0013C, Shanghai, China) containing 1 mmol/L phenylmethyl sulfonyl fluoride (PMSF; Sangon Biotech, A610173, Shanghai, China), and 1 mmol/L phosphate inhibitor cocktail (CWBIO, CW2030, Beijing, China). Protein concentrations were determined using the BCA protein quantification kit (Beyotime). The protein lysates were subjected to sodium dodecyl sulfate-polyacrylamide gel electrophoresis (SDS-PAGE) to separate protein bands, and then they were transferred to a PVDF membrane. After being blocked with 5% skimmed milk for 2 h, the membranes were incubated with primary antibodies at 4 °C overnight. The next day, the membranes were incubated with a relative HRP-conjugated secondary antibody for 1 h at RT. The protein bands on the membrane were visualized using a chemiluminescence imaging system (Tanon, 5200, Shanghai, China). Image J software (NIH, v1.8.0.112, MD, USA) was used to quantify the normalized expressions of those protein bands.

The primary antibodies used above were ZO-1 (1:5000, Proteintech), TLR4 (1:500, Santa Cruz, sc-293072, CA, USA), NLRP3 (1:1000, Adipogen), pro-caspase-1/caspase-1 (1:1000, Adipogen), p-NF-*κ*B p65 (1:500, Santa Cruz, sc-166748, CA, USA), and NF-*κ*B p65 (1:500, Santa Cruz, sc-8008, CA, USA).

### 2.27 Molecular docking

The 3D structure of the target protein TLR4 was retrieved from the Protein Data Bank (PDB) database (https://www.rcsb.org). PyMOL software (Schrödinger, v3.1.0a0, NY, USA) was then employed to perform protein preparation steps, including removal of water molecules, addition of hydrogen atoms, and elimination of native ligands from the receptor structure. The mol2 file of the active compound Son was subsequently downloaded from the PubChem database (https://pubchem.ncbi.nlm.nih.gov) and converted to the pdbqt format using AutoDockTools software (Scripps Research Institute, v1.5.7, CA, USA). Finally, molecular docking simulations were conducted using AutoDock Vina software (Scripps Research Institute, v1.2.5, CA, USA) to evaluate the interaction between Son and TLR4 protein.

### 2.28 Statistical analysis

All experimental data are analyzed as mean ± standard deviation (SD). Statistical analysis was performed by using a two-sample *t*-test. The GraphPad Prism (GraphPad, v9, CA, USA) was used to produce statistical figures. The differences between the two groups were statistically significant when the *P*-value <0.05.

## 3 Results and discussion

### 3.1 Preparation and characterization of Son-lipo

**Figure 1:**
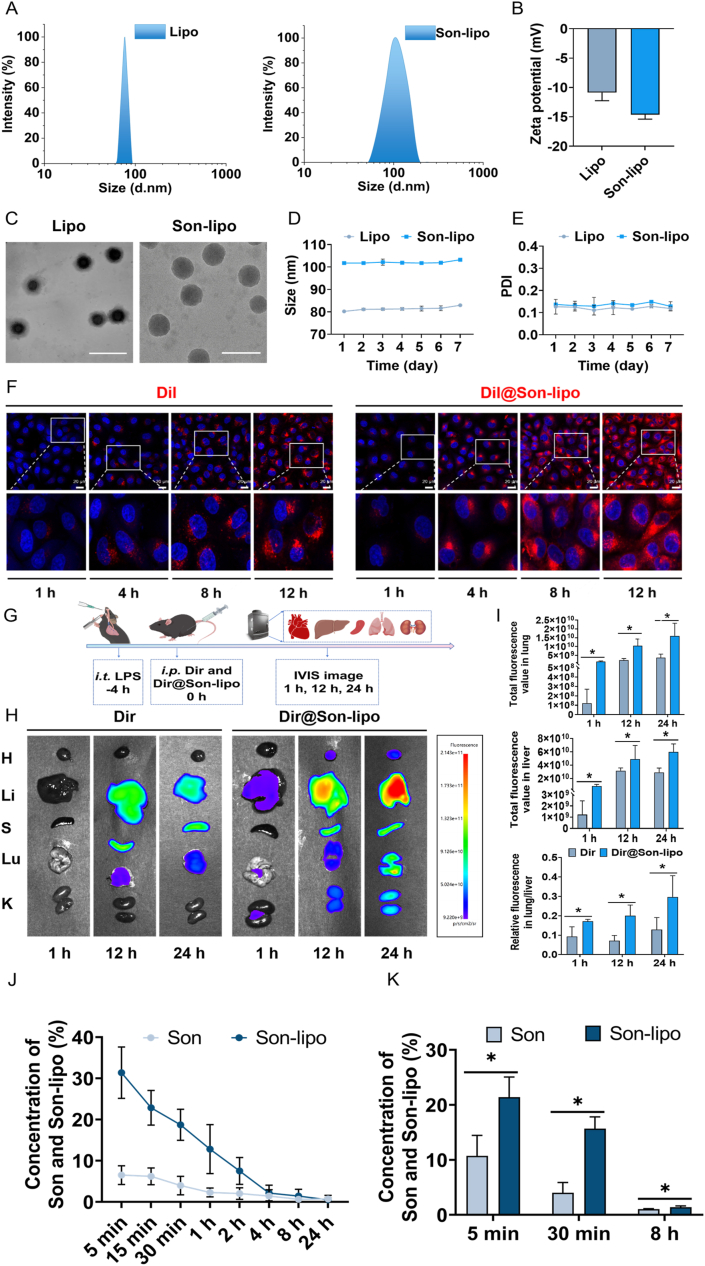
Characterization and biodistribution of Son-lipo. (A) Size distributions, (B) Zeta potential (*n =* 3), and (C) TEM images of Lipo and Son-lipo. Scale bar = 200 nm. The (D) size and (E) PDI of Lipo and Son-lipo in stability test (*n =* 3). (F) The cellular uptake of Son-lipo in LPS-stimulated HUVEC cells. Scale bar = 20 μm. (G) The biodistribution of Dir and Dir@Son-lipo in ARDS mice. (H) Representative *ex vivo* imaging of major organs from each group at 1, 12, and 24 h administration. (I) The distribution of fluorescence in lung, liver, and lung/liver from each group (*n =* 5). (J) Concentration–time curves of Son and Son-lipo in mouse plasma (*n =* 4‒6). (K) Biodistribution of Son and Son-lipo in lung tissues evaluated through LC‒MS/MS at different time points (*n =* 4‒6). Data are represented as mean ± SD. ∗*P* < 0.05.

In this study, to address the obstacles of poor water solubility and low bioavailability of Son, it was intercalated in the DPPC liposomes, referred to as Son-lipo. We continuously optimized Son-lipo by adjusting the ratio of drug to lipids. When this ratio was 1:5, Son lipo had the highest encapsulation efficiency and drug loading capacity, which were 77.5% and 12.5%, respectively (Supporting Information Table S2). To determine the size of both Lipo and Son-lipo, samples were diluted with ddH_2_O at a 1:20 ratio. Dynamic light scattering (DLS) results indicated that the size of the Lipo was about 80.21 nm, while Son-lipo measured approximately 102 nm (Fig. 1A). Additionally, the zeta potential of Son-lipo was −15.07 mV (Fig. 1B). TEM images (Fig. 1C) revealed that both Lipo and Son-lipo exhibited a spherical vesicle structure, consistent with the hydrodynamic size. To assess the stability of Lipo and Son-lipo, the size (Fig. 1D) and PDI (Fig. 1E) were monitored over a period of 7 consecutive days. The results showed that the sizes of both remained stable at approximately 81 and 102 nm, respectively, with a PDI of around 0.13 for both.

In order to investigate the cellular uptake behavior of Son-lipo, fluorescence microscopy was employed to capture images of Dil-labeled (red) Son-lipo at various periods in HUVEC (Fig. 1F and Supporting Information Fig. S1A), MH-S (Fig. S1B), and RAW264.7 (Supporting Information Fig. S2) cells. Compared to the free Dil group, the Dil@Son-lipo group exhibited significantly higher and uniform fluorescence intensity, and the variation in uptake between different cell types was minimized, demonstrating the favorable cellular uptake characteristics of Son-lipo.

Accurate pulmonary targeting is crucial for enhancing drug effects in ARDS. To explore the lung targeting potential of Son-lipo *in vivo*, we embedded the fluorescence probe Dir (red) to label Son-lipo, referred to as Dir@Son-lipo. The organ distribution images at different periods were captured using a IVIS (Fig. 1G). As illustrated, Dir@Son-lipo exhibited a higher fluorescent signal in lung tissue compared to free Dir (Fig. 1H). Over time, the accumulation of Dir@Son-lipo gradually increased in the lungs, leading to a rising fluorescence ratio between the lungs and liver (Fig. 1I). This confirmed that Son-lipo displayed lung targeting capability and favorable distribution in the lungs, providing a promising strategy for the precise targeted treatment of ARDS.

To further elucidate the distribution of Son-lipo *in vivo*, pharmacokinetic studies of Son and Son-lipo were conducted in mice following nebulized inhalation. Plasma concentrations of both drugs were quantified by LC–MS/MS. The results demonstrated that the half-life of Son-lipo was approximately 2.5-fold longer than that of free Son, accompanied by an increased area under the curve (AUC) and a 2.0-fold reduction in clearance (Table 1 and Fig. 1J). These findings indicated an improved pharmacokinetic profile for Son-lipo, suggesting its potential as a sustained-release formulation.

**Table 1:** Pharmacokinetic data of Son/Son-lipo after nebulized inhalation.

Pharmacokinetic parameters	Son	Son-lipo
*C*_max_ (mg/L)	14.78 ± 6.68	55.48 ± 12.82∗
*T*_max_ (h)	0.45 ± 0.11	0.15 ± 0.09∗
*t*_1/2_ (h)	3.34 ± 1.61	8.03 ± 1.29∗
AUC_0‒∞_ (h**·**ng/mL)	19.06 ± 4.50	36.96 ± 12.43∗
MRT_0‒∞_ (h)	4.24 ± 2.42	8.00 ± 2.01∗
AUC_last_ (h**·**ng/mL)	22.69 ± 8.54	31.96 ± 4.49∗

Notes: *C*_max_, the maximum plasma concentration; *T*_max_, the time to reach this peak; *t*_1/2_, the elimination half-life; AUC_0‒∞_, the area under the concentration-time curve; MRT_0‒∞_, to the mean residence time from administration until complete elimination; AUC_last_, the area under the concentration–time curve from administration to the time of the last measurable drug concentration; Son, Songorine; Son-lipo, Son@liposome. Data are presented as mean ± SD (*n* = 4‒5). ∗*P* < 0.05, compared to the Son.

Subsequently, the pulmonary biodistribution of Son and Son-lipo was evaluated at 5, 30 min, and 8 h after drug nebulized inhalation. The results showed the concentration of Son-lipo in lungs was significantly higher than that of Son (Fig. 1K), indicating enhanced pulmonary distribution.

Son-lipo's lung-targeting property is mainly attributed to the DPPC, a lung surfactant^43^. Liposomes containing DPPC are more readily recognized and internalized by alveolar cells, demonstrating enhanced lung-targeting capability^44^^,^^45^. Furthermore, the balanced hydrophilic–lipophilic characteristics of DPPC liposomes allow stable dispersion in the alveolar fluid, promoting uniform pulmonary distribution and effective deposition of the loaded drugs, thereby augmenting local therapeutic efficacy^46^^,^^47^.

The above characterization results highlight the successful preparation of Son-lipo and demonstrate its remarkable stability and lung-targeting property. These findings underscore its potential efficacy in the treatment of ARDS.

### 3.2 Biosafety of Son-lipo *in vitro* and *in vivo*

**Figure 2:**
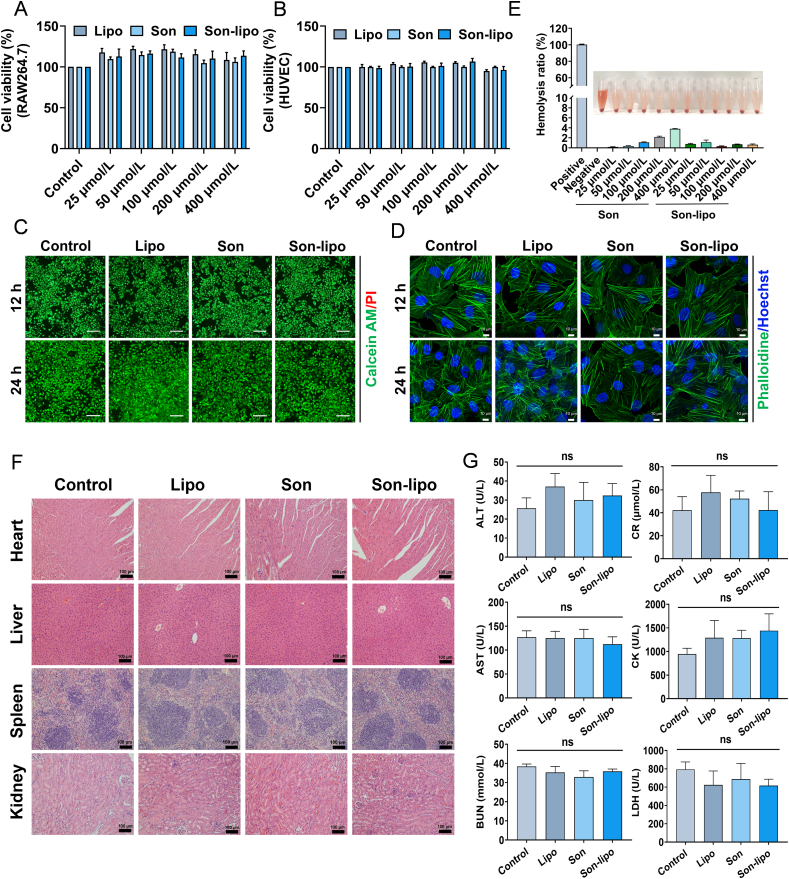
The biosafety of Son-lipo *in vitro* and *in vivo*. (A, B) The cytotoxicity of Lipo, Son, and Son-lipo in RAW264.7 and HUVEC cells (*n =* 3). (C) Live/dead staining. (Scale bar = 100 μm) and (D) Cytoskeleton staining (Scale bar = 10 μm) of HUVEC cells after incubation with Lipo, Son, and Son-lipo for 24 h. (E) Hemolysis ratio assays (*n =* 3). (F) *In vivo* safety evaluation of the drugs on other major organs, including heart, liver, spleen, and kidney tissues in ARDS mice by HE staining. Scale bar = 100 μm. (G) The biochemical analysis of ALT, CR, AST, CK, BUN, and LDH in blood (*n =* 4‒5). Data are represented as mean ± SD. ns, not significant.

Biosafety is a crucial factor for the clinical implementation of drugs. Our preliminary evaluation assessed the cytotoxic potential of Lipo, Son, and Son-lipo within HUVEC and RAW264.7 cell lines employing the CCK-8 assay. No significant reduction in cell viability was observed across all groups after 12 or 24 h of treatment with the three drugs (Fig. 2A and B). Under toxic conditions, the cytoskeletal structure may contract, fragment, or be completely lost. Therefore, we further evaluated the cytotoxic effects of the drugs on HUVEC cells by employing live/dead cell assays and cytoskeletal staining techniques. After 12 and 24 h of treatment, the proportion of viable cells, as indicated by calcein AM staining (green), approached nearly 100%, whereas propidium iodide (PI) staining (red) marked only a negligible number of dead cells (Fig. 2C and Supporting Information Fig. S3A and S3B). Moreover, no significant contraction or disappearance of the cytoskeleton was induced by any of the tested drugs (Fig. 2D, Fig. S3C and S3D). These findings substantiated the favorable biosafety of Son-lipo *in vitro*.

Furthermore, hemolysis is associated with various immune and genetic complications, and certain drugs have been known to induce hemolytic reactions^48^. Hence, the compatibility of the drugs with rat erythrocytes at concentrations ranging from 25 to 400 μmol/L was evaluated. The coloration of all drug-treated groups was similar to that of the Control, showing a hemolysis rate of less than 4% (Fig. 2E), demonstrating that neither Son nor Son-lipo induced hemolysis. Subsequently, to assess the biosafety of the three drugs *in vivo*, major organs (heart, liver, spleen, and kidney) were harvested from ARDS mice for HE staining. The observations revealed (Fig. 2F) that none of the experimental groups demonstrated notable tissue damage. RT-qPCR analysis further corroborated that there were no significant differences in the expression levels of inflammatory cytokines among the various groups across different tissues (Supporting Information Fig. S4), indicating the absence of discernible organ lesions following extended nebulized administration.

To further quantitatively substantiate the safety profile of the drugs, blood samples from the mice were collected for blood biochemical and blood routine examination. All measured indicators were within normal ranges (Fig. 2G), including alanine aminotransferase (ALT), aspartate aminotransferase (AST), creatine kinase (CK), lactate dehydrogenase (LDH), creatinine (CR), and blood urea nitrogen (BUN). Furthermore, inflammatory markers, such as lymphocyte count (Lym#), neutrophil count (GR#), lymphocyte percentage (Lym%), and neutrophil percentage (GR%), were not significantly abnormal (Supporting Information Fig. S5). These findings underscored that Son-lipo exhibited robust biosafety with respect to both blood and tissues.

### 3.3 Antioxidant, endothelial barrier protection, and anti-inflammatory effects of Son-lipo *in vitro*

**Figure 3:**
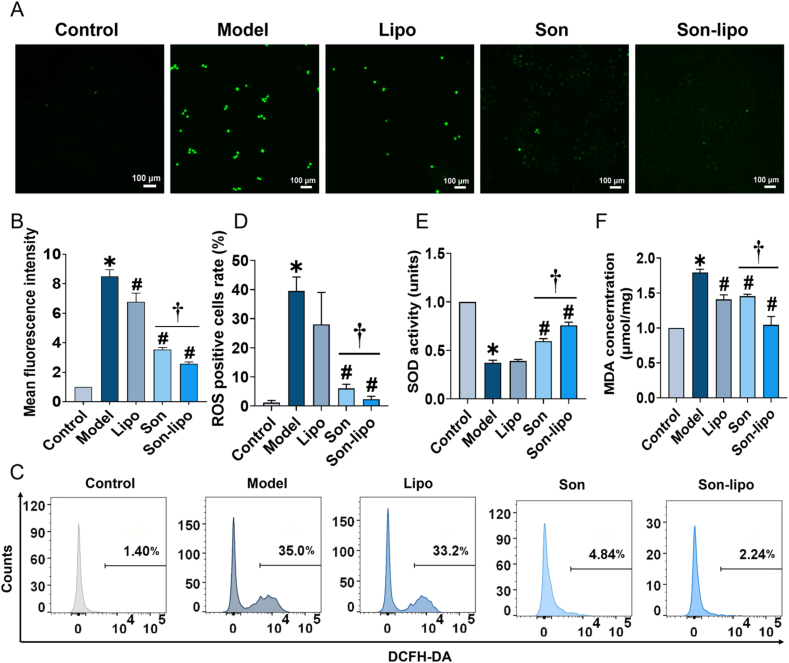
Anti-antioxidant activities of Son-lipo *in vitro*. (A) Fluorescent images (Scale bar = 100 μm) and (B) statistics analysis of intracellular ROS levels in HUVEC cells (*n =* 3). (C) The flow cytometry assay and (D) the DCFH-DA positive cell ratio analysis in HUVEC cells (*n =* 3). Quantification of (E) SOD and (F) MDA in HUVEC cells (*n =* 3). Data are represented as mean ± SD. ∗*P* < 0.05 *vs* Control. ^#^*P* < 0.05 *vs* Model. ^†^*P* < 0.05.

Previous research has established a strong association between ARDS and uncontrolled ROS, which play a crucial role in lung damage^49^^,^^50^. To assess the protective capabilities of Son-lipo against oxidative stress in HUVEC cells, fluorescent probe DCFH-DA staining was conducted to examine ROS levels within the cells. The results (Fig. 3A) and subsequent quantification (Fig. 3B) revealed a marked decrease in fluorescence intensity following Son-lipo treatment, indicative of diminished intracellular ROS production. This finding was consistent with the flow cytometry analysis (Fig. 3C and D), both experiments substantiated the role of Son-lipo in mitigating oxidative stress.

Intracellular superoxide dismutase (SOD) plays a pivotal role in preserving cellular redox balance by scavenging ROS, and serves as an indicator of the cellular antioxidant status. Consequently, we evaluated SOD levels in HUVEC cells challenged by LPS. It was found that Son and Son-lipo curtailed the decline of SOD levels, signifying their efficacy in safeguarding against oxidative stress-induced damage (Fig. 3E). Remarkably, Son-lipo demonstrated the most pronounced protective effect. Besides, malondialdehyde (MDA) analysis further corroborated that Son-lipo protected cells from lipid peroxidation damage through ROS scavenging (Fig. 3F). Collectively, these results highlighted the exceptional antioxidant capacity of Son-lipo *in vitro*.

**Figure 4:**
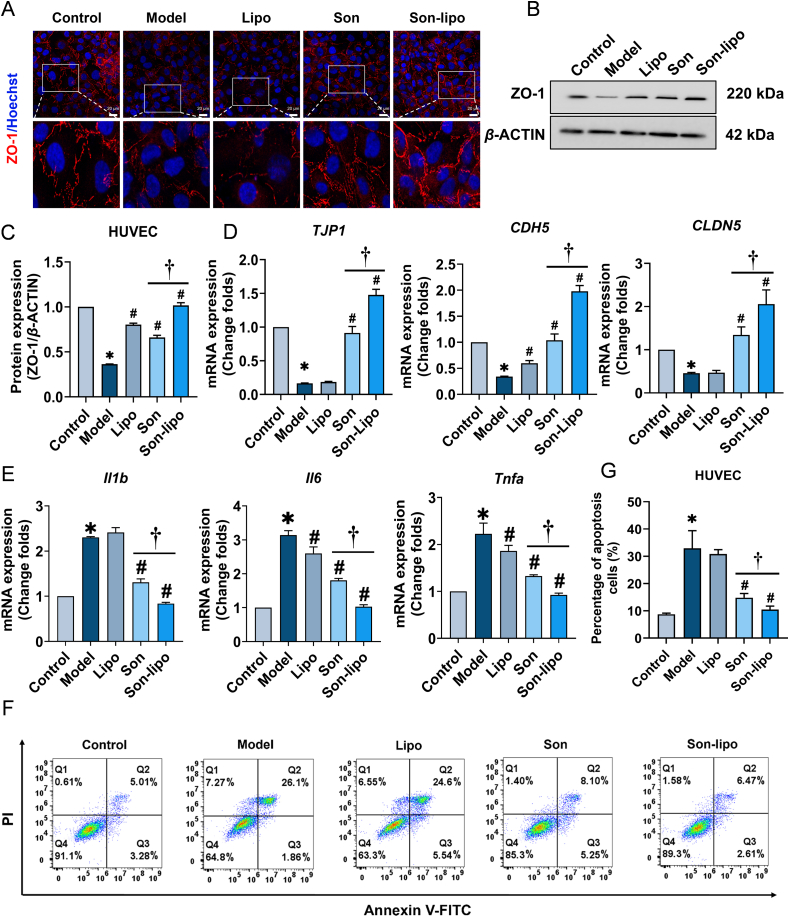
Effects of Son-lipo in repairing the endothelial barrier, inhibiting inflammation, and mitigating cell apoptosis *in vitro*. (A) Immunofluorescent staining of tight junction factor ZO-1 in HUVEC cells treated with different formulations. Red, ZO-1. Blue, Hoechst33342. Scale bar = 20 μm. Protein expression of ZO-1 by (B) Western blot and (C) statistical analysis in HUVEC cells (*n =* 3). (D) The mRNA expression of tight junction factors, *TJP1*, *CDH5*, and *CLDN5*, in HUVEC cells by RT-qPCR (*n =* 3). (E) The mRNA expression of inflammatory factors, *Il1b*, *Il6,* and *Tnfa*, in RAW264.7 cells by RT-qPCR (*n =* 4). Internal control, *α*-Tubulin. The apoptosis rate of endothelial cells was detected by (F) flow cytometry and (G) analyzed by FlowJo in HUVEC cells (*n =* 3). Data are represented as mean ± SD. ∗*P* < 0.05 *vs* Control. ^#^*P* < 0.05 *vs* Model. ^†^*P* < 0.05.

Disruption of the endothelial barrier is a key characteristic of ARDS^51^. To examine the protective efficacy of Son-lipo on endothelial barrier integrity, HUVEC cells were employed to analyze the expression levels of the tight junction protein ZO-1. Immunofluorescence (red) revealed a disruption of ZO-1 protein following LPS challenge. Treatment with Son and Son-lipo restored the disrupted red fluorescence, demonstrating that both treatments ameliorated the downregulation and disruption of ZO-1 protein (Fig. 4A and Supporting Information Fig. S6A). To precisely quantify ZO-1 expression, Western blot analysis was conducted on HUVEC cells stimulated with LPS. The findings indicated that both Son and Son-lipo mitigated the downregulation of ZO-1 protein, with Son-lipo exhibiting greater efficacy (Fig. 4B and C). Furthermore, RT-qPCR results showed that Son-lipo enhanced the levels of *TJP1*, *CDH5*, and *CLDN5* (Fig. 4D). Son-lipo also demonstrated superior efficacy in promoting wound healing than free Son (Fig. S6B and S6C). These results provided novel evidence that Son-lipo could effectively repair endothelial barrier dysfunction.

The compromise of the endothelial barrier is frequently accompanied by substantial infiltration of inflammatory cells, which triggers and exacerbates an inflammatory response, leading to massive apoptosis and further damage to the endothelial barrier. To evaluate the impact of Son-lipo on critical inflammatory mediators, we employed an LPS-induced inflammation model using RAW264.7 cells. As expected, both Son and Son-lipo significantly suppressed the expression of these inflammatory mediators (Fig. 4E). In addition, LPS induces oxidative stress and excessive inflammatory responses in cells, ultimately culminating in apoptosis^52^, ^53^, ^54^. Flow cytometry results revealed that the apoptosis rate reached nearly 30% following stimulation with 2 μg/mL LPS for 24 h, but was approximately reduced to 9% after treatment with Son-lipo (Fig. 4F and G). Subsequent gene expression analysis and terminal deoxynucleotidyl transferase-mediated dUTP nick end labeling (TUNEL) staining further substantiated the anti-apoptotic properties of Son-lipo (Fig. S6D and S6E).

In conclusion, Son-lipo mitigates oxidative stress, restores the barrier function, and reduces inflammatory and apoptotic markers *in vitro*, highlighting its considerable potential as a therapeutic agent in the treatment of ARDS.

### 3.4 Pharmacological effects of Son-lipo on the sepsis mouse model

**Figure 5:**
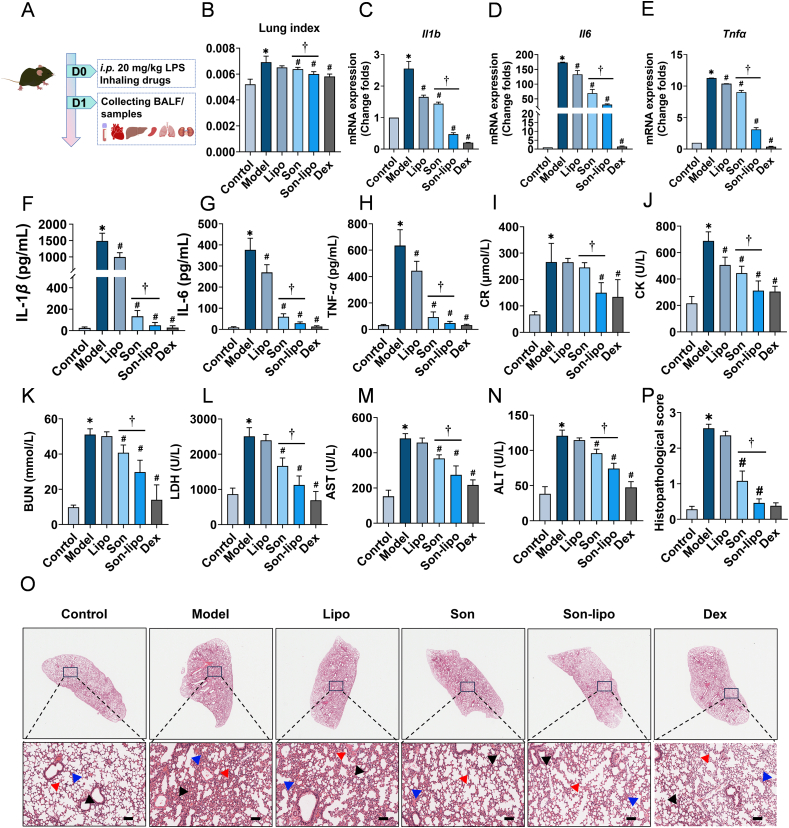
Son-lipo ameliorated the phenotypes of sepsis-induced lung injury in mice. (A) Schedule of the construction of sepsis mouse model, drug administration, and sample collection. (B) Lung index of mice (*n =* 5‒6). (C‒E) The mRNA expressions of inflammatory factors, *Il1b*, *Il6*, and *Tnfa*, in lung tissues by RT-qPCR (*n =* 4). Internal control, *α*-Tubulin. (F‒H) The ELISA analysis of serum IL-1*β*, IL-6, and TNF-*α* levels (*n =* 4‒5). (I‒N) The biochemical analysis of CR, CK, BUN, LDH, AST, and ALT in blood (*n =* 5‒6). (O) HE staining of lung tissues. Black arrows, the degree of inflammatory cell infiltration and destroyed alveoli structures. Red arrows, alveoli wall. Blue arrows, the degree of alveoli leakage. Scale bar = 1000 μm for the upper panels and 100 μm for the lower panels. (P) Pathological scores of HE staining results (*n =* 5). Data are represented as mean ± SD. ∗*P* < 0.05 *vs* Control. ^#^*P* < 0.05 *vs* Model. ^†^*P* < 0.05.

To investigate the therapeutic efficacy of Son-lipo in sepsis mice, the experimental model was established by intraperitoneal injection of 20 mg/kg LPS. Son and Son-lipo were administered *via* nebulized inhalation, followed by collection of blood and tissue samples for comprehensive analysis (Fig. 5A). Son and Son-lipo were found to significantly reduce lung index in sepsis mice compared to the Model group (Fig. 5B), indicating decreased lung edema. Besides, compared to the Model group, both treatments downregulated the mRNA expressions of pro-inflammatory factors (*Il1b*, *Il6,* and *Tnfa*), with Son-lipo showing a superior effect to Son (Fig. 5C‒E). These results revealed that Son-lipo significantly relieved alveolar edema and inflammation, suggesting an improvement of the pulmonary inflammatory environment in sepsis mice.

To further evaluate the therapeutic effect, serum IL-1*β*, IL-6, and TNF-*α* were measured by ELISA. As expected, Son-lipo treatment significantly reversed the upregulation of these cytokines in the Model group (Fig. 5F‒H). Subsequently, blood samples were collected for biochemical analyses. All parameters were significantly elevated in the Model group but decreased following treatments, with the most pronounced reduction observed in the Son-lipo group (Fig. 5I‒N).

HE staining (Fig. 5O) and histopathological scoring (Fig. 5P) were performed to assess the degree of lung injury. The Model group exhibited extensive infiltration of inflammatory cells, accompanied by disrupted alveolar architecture and thickening of the alveolar walls compared to the Control group. In contrast, Son-lipo treatment resulted in substantially diminished inflammatory infiltration (indicated by black arrows), reconstruction of alveolar structure (blue arrows), and thinning of the alveolar septa (red arrows) in comparison with those observed in the Model group. These results underscored a progressive attenuation of pulmonary injury in sepsis mice following Son-lipo treatment.

HE staining of the heart, liver, spleen, and kidney revealed pronounced structural damage and inflammatory cell infiltration in the Model group, whereas Son-lipo treatment, to some extent, reversed these pathological changes (Supporting Information Fig. S7).

Taken together, these results emphasized the efficacy of Son-lipo in ameliorating lung injury induced by sepsis in mice.

### 3.5 Therapeutic effects of Son-lipo on the ARDS mouse model

**Figure 6:**
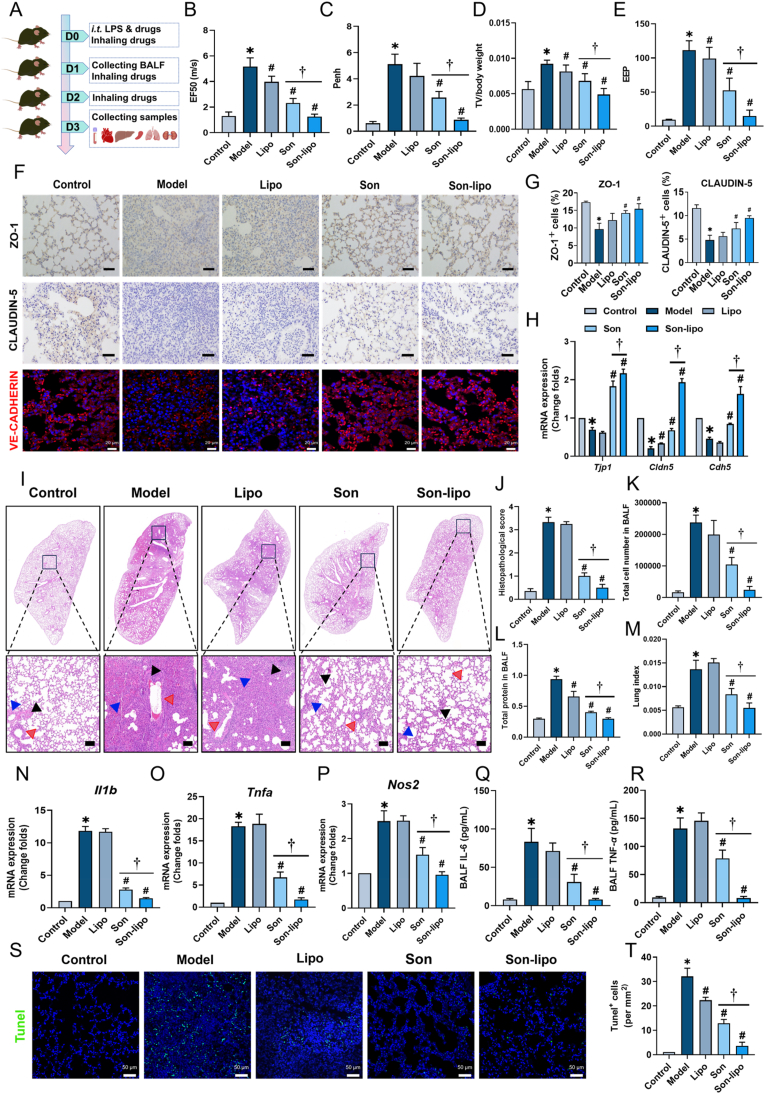
Son-lipo ameliorated the phenotypes of LPS-induced ARDS *in vivo*. (A) Schedule of the construction and treatments of ARDS mouse model. (B‒E) The results of pulmonary function indexes (EF50, Penh, TV/body weight, and EEP) by non-invasive whole-body plethysmography (*n =* 4‒5). (F) Immunohistochemistry staining of ZO-1 and CLAUDIN-5 protein (Scale bar = 100 μm), and immunofluorescent staining of VE-CADHERIN protein (red, VE-CADHERIN; blue, Hoechst33342; Scale bar = 20 μm). (G) Statistical analysis of ZO-1 and CLAUDIN-5 protein expression in F (*n =* 3). (H) The mRNA expressions of tight junction factors, *Tjp1*, *Cldn5*, and, *Cdh5* in lung tissues by RT-qPCR analysis (*n =* 3). Internal control, *α*-Tubulin. (I) HE staining of lung tissues. Black arrows, the degree of inflammatory cell infiltration and destroyed alveoli structures. Red arrows, alveoli wall. Blue arrows, the degree of alveoli leakage. Scale bar = 1000 μm for the upper panels and 100 μm for the lower panels. (J) Pathological scores of HE staining results (*n =* 5‒6). (K) Total cell number and (L) protein content in BALF (*n =* 4‒5). (M) Lung index of mice (*n =* 5). (N‒P) The change in folds of mRNA levels of inflammatory factors, *Il1b*, *Tnfa*, and *Nos2*, in lung tissues (*n =* 3). (Q, R) The ELISA analysis of IL-6 and TNF-*α* levels (*n =* 4). (S) TUNEL staining and (T) statistics of positive cells in lung tissues (*n =* 3). Blue, Hoechst33342. Scale bar = 50 μm. Data are represented as mean ± SD. ∗*P* < 0.05 *vs* Control. ^*#*^*P* < 0.05 *vs* Model. ^†^*P* < 0.05.

To better demonstrate the prominent efficacy of Son-lipo in mitigating oxidative stress, preserving endothelial integrity, and curbing inflammation and apoptosis, the therapeutic potential of its nebulized inhalation was investigated in an ARDS mouse model established by tracheal injection of LPS. After three days of drug inhalation, pulmonary function was assessed, alongside the collection of lung tissues for comprehensive analysis (Fig. 6A). Key pulmonary function indicators such as EF50, Penh, TV/Body, and EEP were measured using the noninvasive whole-body plethysmography method. The results demonstrated a significant restoration of lung function following treatment with Son-lipo (Fig. 6B‒E).

Given that damage to the pulmonary vascular endothelium is a principal contributor to pulmonary edema and heightened lung inflammation, the therapeutic impact of Son-lipo on endothelial barrier disruption was assessed in an ARDS mouse model. Immunohistochemical and Immunofluorescent staining analyses disclosed that upon LPS stimulation, both the Model and Lipo groups exhibited diminished positive areas for ZO-1, CLAUDIN-5, and VE-CADHERIN. In contrast, positive regions for these proteins increased after treatment with Son or Son-lipo (Fig. 6F‒G). What's more, the gene expression levels of these tight junction markers, which decreased following LPS stimulation, were reversed by Son and Son-lipo (Fig. 6H). These findings implied that Son-lipo possessed the capability to repair endothelial barrier damage *in vivo*.

Endothelial barrier damage leads to the pervasive leakage of inflammatory mediators, thereby intensifying the severity of lung injury. Consequently, evaluating the inflammatory status is vital for forecasting the extent of pulmonary damage. We initiated our investigation by HE staining (Fig. 6I) and conducted pathological scoring (Fig. 6J). The Model group manifested the most extensive infiltration of inflammatory cells, accompanied by significant destruction of alveolar structures and blood extravasation. In contrast, Son-lipo effectively reinstated the integrity of the damaged alveolar architecture (denoted by black arrows), alleviated alveolar epithelial injury (denoted by blue arrows), and reduced alveolar wall thickening (denoted by red arrows). These results highlighted that the inflammatory response in ARDS mice progressively diminished following Son-lipo treatment. To further validate these results, BALF was collected to assess total cell counts and protein concentration. Following LPS stimulation, both cell numbers and protein concentration increased, while treatments with Son and Son-lipo mitigated these increases (Fig. 6K and L).

The lung index serves as an indicator of the severity of pulmonary edema. We discovered that both Son and Son-lipo significantly reduced the lung index in ARDS mice (Fig. 6M). Consistent with the *in vitro* experimental results, Son and Son-lipo treatments decreased pro-inflammatory factors, including *Il1b*, *Tnfa*, and *Nos2* (Fig. 6N‒P), and *Il6* (Supporting Information Fig. S8), with the Son-lipo group showing the most pronounced effect. The findings revealed that Son-lipo effectively downregulated the overexpression of these genes, indicating an improvement in the inflammatory status of murine lungs. To further assess the therapeutic outcomes, the expression levels of IL-6 and TNF-*α* in BALF were determined by ELISA. As anticipated, treatment with Son-lipo resulted in a marked reduction in IL-6 and TNF-*α* levels (Fig. 6Q and R).

Excessive inflammatory responses can escalate the rate of cell apoptosis in lung tissues. To evaluate this, TUNEL staining was conducted in lung tissues. In the Model group, there was a marked increase in the number of positively stained apoptotic cells (green), a condition that was effectively ameliorated by treatment with Son and Son-lipo (Fig. 6S and T). To further substantiate these findings, we quantified the mRNA levels of two crucial apoptosis markers, *Bax* and *Bcl2*, and calculated their respective ratio. Our analysis revealed that Son-lipo treatment downregulated *Bax* expression and concomitantly upregulated *Bcl2* transcripts, indicating that Son-lipo proficiently impedes apoptosis (Supporting Information Fig. S9).

In summary, Son-lipo significantly improves multiple phenotypes of ARDS mice: it can enhance lung function, reduce lung injury, maintain pulmonary endothelial cell integrity, suppress pulmonary inflammation, and inhibit apoptosis. These multifaceted therapeutic effects suggest that Son-lipo holds promise as an effective nanomedicine for treating ARDS.

### 3.6 Overall effects of Son-lipo on ARDS by RNA-seq *in vivo*

**Figure 7:**
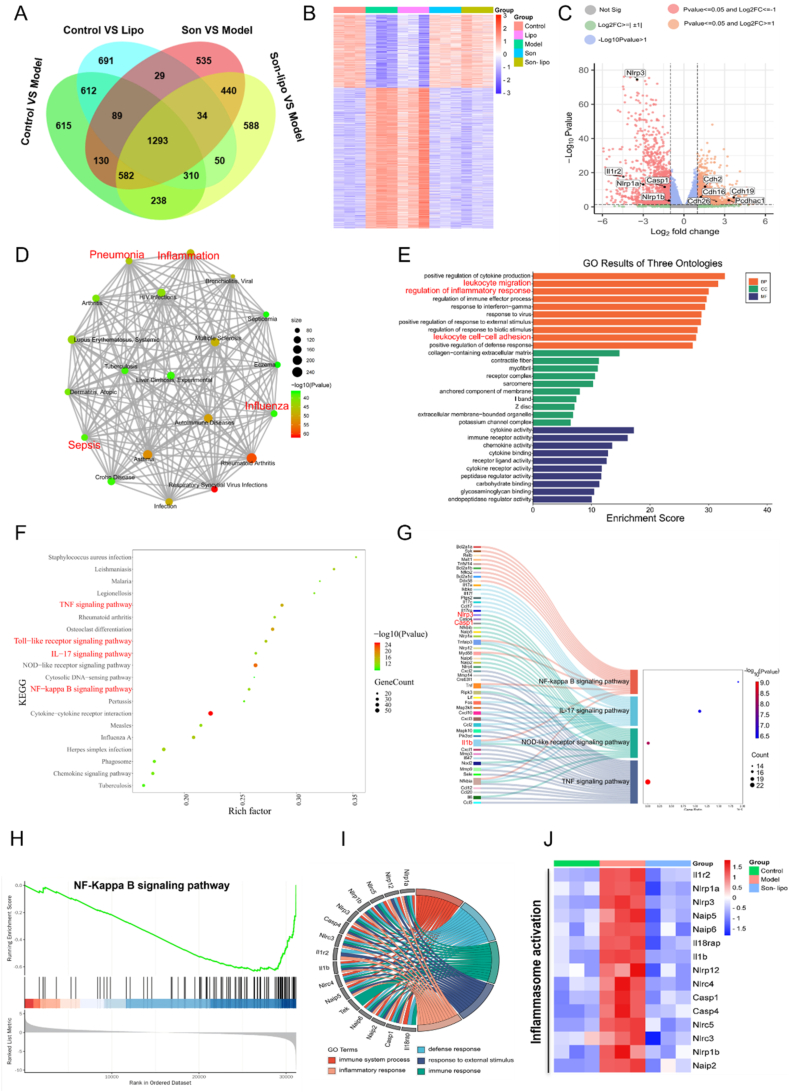
RNA-seq analysis of the overall effects of Son-lipo for ARDS in lung tissues. (A) Venn diagram of intersections of DEGs among different comparisons, including Control and Model, Control and Lipo, Son and Model, and Son-lipo and Model. (B) Heatmap analysis of 1293 shared DEGs in the venn diagram. (C) Volcano plot of the DEGs between the Model and Son-lipo groups. Red points on the left represented downregulated DEGs in Son-lipo. In contrast, orange points on the right represented upregulated ones. The upregulated DEGs in the Model were subjected to (D) disease enrichment analysis, (E) GO analysis in a bar plot, and (F) KEGG analysis in a bubble diagram. (G) Sankey dot pathway enrichment between Son-lipo and Model. (H) GSEA plot of hub genes in NF-*κ*B signaling pathway. (I) GO chord diagram of genes associated with inflammasome activation. (J) Cluster analysis of key DEGs in NF-*κ*B/NLRP3 signaling pathway.

To elucidate the overall effects of Son-lipo on ARDS, RNA-Seq was employed to analyze the transcriptomic profile alterations in murine lung tissues across all groups. The differentially expressed genes (DEGs) between various comparisons—Control and ARDS Model, Control and Lipo, Son and Model, and Son-lipo and Model—were intersected and illustrated as a Venn diagram, revealing 1293 shared DEGs (Fig. 7A). Heat mapping of all shared DEGs demonstrated that gene expression levels in Son/Son-lipo resembled those in the Control, but the Model and Lipo exhibited contrasting patterns, indicating substantial recovery of the inflamed lungs. Furthermore, approximately two-thirds of DEGs were downregulated following Son or Son-lipo treatments, reflecting a strong association with ARDS phenotypes (Fig. 7B). In comparison to the Model, volcano maps indicated that DEGs related to inflammasome activation were dramatically downregulated in the Son-lipo group, including *Nlrp3*, *Casp1*, and *Nlrp1a*. Son-lipo also upregulated vascular endothelial cell protection factors, such as *Cdh2*, *Cdh19*, and *Cdh26* in RNA-seq analysis (Fig. 7C).

Those upregulated DEGs in the model but not the Son-lipo group were predominantly associated with respiratory diseases, revealing that Son-lipo exerted a negative regulatory effect on various conditions such as pneumonia, inflammation, influenza, and sepsis (Fig. 7D). Subsequent GO analysis (Son-lipo *vs.* Model) showed enrichment in various immunological and inflammation-related GO terms (Fig. 7E). KEGG analysis further demonstrated that Son-lipo has significant potential to inhibit various inflammatory signals (Fig. 7F), such as the TNF-*α*, NF-*κ*B, toll-like receptor, and IL-17 signaling pathways. These findings corroborate the experimental outcomes, suggesting that Son-lipo can effectively modulate inflammatory responses. Similar results were observed in the shared upregulated and downregulated DEGs in the Model as compared to the Control, Son, and Son-lipo groups (Supporting Information Figs. S10 and S11).

Additionally, those downregulated DEGs (Son-lipo *vs.* Model) revealed significant associations with hemophilia and hypertensive disorders (Supporting Information Fig. S12). This suggests that Son-lipo may not be suitable for treating certain hematologic conditions. When comparing the effects of Son-lipo and Son alone, disease analysis indicated that the upregulated DEGs in the Son group were enriched in various inflammation-related diseases, such as sepsis (Supporting Information Fig. S13), opposite with those of the downregulated ones (Supporting Information Fig. S14A‒S14D). The results of the GO and KEGG analyses also indicated that the anti-inflammatory effect of Son-lipo may be superior to that of Son.

To investigate the protective effect of Son-lipo on vascular endothelium, a heat map study of critical indicators of the endothelial barrier was conducted. The finding showed that factors associated with a protective endothelium barrier were down-regulated in the Model group but upregulated in both the Control and Son-lipo (Fig. S14E). Notably, accumulating evidence suggests that NLRP3 inflammasome activation is an important event in the development of ARDS^55^, ^56^, ^57^, ^58^. Sankey dot pathway enrichment results (Fig. 7G) displayed that DEGs were closely associated with *Nlrp3*, *Casp1*, *Il1b,* and other key inflammation activation factors. These DEGs were also enriched in several inflammatory signaling pathways, including the IL-17, NF-*κ*B (Fig. 7H), and NOD-like receptor signaling pathways. The GO chord diagram of DEGs (Fig. 7I) further supported these findings. Many DEGs related to NF-*κ*B/NLRP3 signaling pathway were up-regulated in the Model and down-regulated in the Control and Son-lipo (Fig. 7J), suggesting that Son-lipo might prevent the activation of this pathway.

In conclusion, Son-lipo is found to downregulate multiple pro-inflammatory signaling pathways and inhibit NLRP3 inflammasome activation, demonstrating significant therapeutic potential for various pulmonary inflammatory disorders, including ARDS.

### 3.7 Anti-inflammatory mechanism of Son-lipo *in vivo*

The TLR4/NF-*κ*B/NLRP3 pathway is intricately linked to the activation of the inflammatory response during ARDS^56^^,^^59^. It plays a crucial role in exacerbating inflammation during systemic infections or other illnesses. Based on RNA-seq results, further experiments were conducted to verify that Son and Son-lipo might exert anti-inflammatory effects by inhibiting the activation of TLR4/NF-*κ*B/NLRP3 signaling pathway.

**Figure 8:**
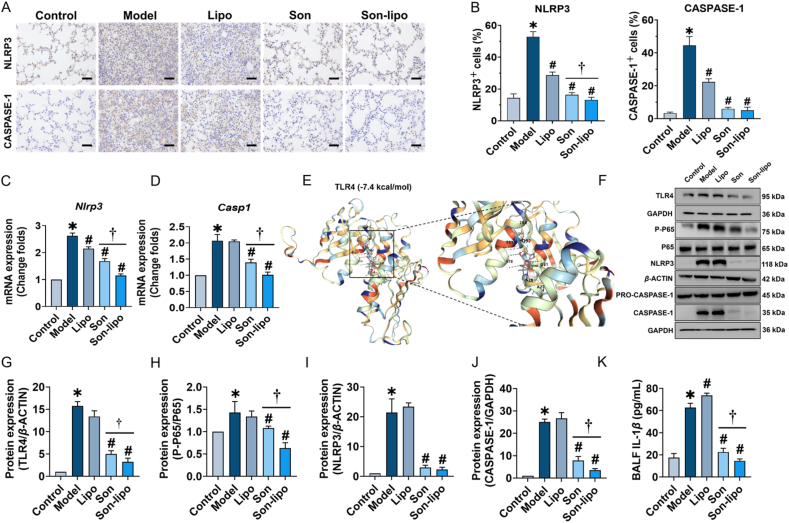
Son-lipo inhibited the activation of NLRP3 inflammasome. (A) The immunohistochemistry staining and (B) statistical analysis of NLRP3 and CASPASE-1 proteins (*n =* 3). Scale bar = 100 μm. (C, D) The *Nlrp3* and *Casp1* transcripts in lung tissues by RT-qPCR (*n =* 3). Internal control, *α*-Tubulin. (E) The binding activity between Son and TLR4 protein was shown by an *in silico* molecular docking analysis. (F) The protein expressions of TLR4, P-P65, P65, NLRP3, PRO-CASPASE-1, and CASPASE-1 in the lung tissues by Western blot and (G‒J) their statistical analysis (*n =* 3). (K) Expression levels of IL-1*β* by ELISA (*n =* 4). Data are represented as mean ± SD. ∗*P* < 0.05 *vs* Control. ^*#*^*P* < 0.05 *vs* Model. ^†^*P* < 0.05.

Firstly, the immunohistochemistry was applied to quantify indicators of inflammasome activation. After LPS treatment, both the Model and Lipo groups exhibited a substantial increase in the positive staining area for CASPASE-1 and NLRP3. In contrast, treatments with Son and Son-lipo significantly reduced the positive areas for these two proteins (Fig. 8A and B). This demonstrated that Son and Son-lipo may suppress the activation of NLRP3 inflammasome, thereby mitigating inflammatory damage. Furthermore, the mRNA expression levels were consistent with the immunohistochemistry findings (Fig. 8C and D), further reinforcing the conclusion that Son-lipo possessed protective effects against inflammation.

Subsequently, molecular docking and Western blot were conducted to further elucidate the mechanisms by which Son-lipo treated ARDS mice. Molecular docking analysis revealed a strong binding affinity between Son and TLR4 (−7.4 kcal/mol) (Fig. 8E). In an ARDS model group, Western blot indicated the expression levels of TLR4, phosphorylated P65 (p-P65), NLRP3, and caspase-1 were notably upregulated compared to the Control group; remarkably, Son-lipo treatment effectively reversed the upregulation of these proteins (Fig. 8F‒J). Moreover, the degree of protein downregulation was more pronounced in the Son-lipo treatment group than in the Son group. On the other hand, Son-lipo effectively reduced the expression of the inflammatory cytokine IL-1*β* in the BALF (Fig. 8K), reaffirming that Son-lipo could mitigate inflammation by inhibiting the activation of NLRP3 inflammasome.

In the pathogenesis of ARDS, LPS is specifically recognized by TLR4, which subsequently promotes the phosphorylation and degradation of *Iκ*B*α*, facilitating the assembly of the NLRP3 inflammasome. This cascade culminates in the maturation and release of caspase-1 (CASP1)-mediated inflammatory cytokines and the induction of apoptosis^60^. Our study found that Son-lipo exerts a negative regulatory effect on the activation of the TLR4/NF-*κ*B/NLRP3 pathway, offering preliminary insights into the mechanism by which Son-lipo mitigates ARDS. Nonetheless, the precise mechanism still requires further investigation. Given the close association of this pathway with various other diseases, such as atherosclerosis^61^ and diabetes^62^, it is valuable to further explore the therapeutic potential of Son-lipo across these conditions.

## 4 Conclusions

In this study, a lung-targeted lipid nanomedicine was developed by intermingling Son in DPPC liposomes (Son@liposome, Son-lipo). First, *in vitro* and *in vivo* experiments confirmed Son-lipo's stable physicochemical properties, good biosafety, lung-targeted ability, anti-inflammatory effects, and protective roles on the endothelial barrier. Then, in an LPS-induced ARDS mouse model, Son-lipo significantly mitigated ARDS pathological phenotypes, including uncontrolled inflammation, pulmonary dysfunction, alveolar structural disruption, and disruption of endothelial integrity. Finally, transcriptome sequencing and Western blotting revealed that Son-lipo exerted its therapeutic effects on ARDS mainly by inhibiting the NLRP3 inflammasome activation. Together, our study demonstrates that the new lipid nanomedicine (Son-lipo) alleviates ARDS through repairing the endothelial barrier and inactivating NLRP3 inflammasome, hence providing a candidate strategy for the clinical treatment of ARDS.

There are also several limitations in this study. Firstly, our study used two representative ARDS animals to clarify Son's therapeutic potential; however, the effects of Son-lipo in ARDS patients have not been investigated due to some ethical restrictions. Secondly, we assess the lung-targeting property of Son-lipo, but which cell type or organelle it can target remains to be clarified for a precise explanation of its functions. Last but not least, the underlying mechanism of Son-lipo treating ARDS mainly focuses on proinflammatory signals, and other key pathways such as HIF-1*α* and VE-Cadherin regulating endothelial function may need in-depth research in the future.

In future research, it is essential to further elucidate the specific mechanisms of Son-lipo in treating ARDS through techniques such as gene editing. Exploring combination therapies that integrate nanomedicine with conventional treatments (*e.g.*, corticosteroids, ventilation strategies) may also yield synergistic benefits.

## Author contributions

Danwen Zheng, Zhongde Zhang, Jun Wu, and Yuntao Liu conceived, supervised and provided funding for the research. Haiyan Wang and Zhi-Chao Sun carried out the experiments and performed data analysis. Haiyan Wang, Zhi-Chao Sun, and Chunlei Dai revised the manuscript. Ran Liao and Ran Lin helped multiple cell experiments. Wenjun Fu provides several guides for study design. Liying Wang and Ruhe Zhang provided some technical assistances and performed TEM experiments. Haiyan Wang wrote the manuscript. All authors reviewed the results and approved the final version of the manuscript.

## Conflicts of interest

The authors declare no conflicts of interest.

## Appendix A Supporting information

The following is the Supporting Information to this article.
