# Supplementary material for: Inhalable songorine-integrated lipid nanomedicine for targeted ARDS therapy via repairing endothelial barrier and inactivating NLRP3 inflammasome
Source: Acta Pharm Sin B. 2025 Nov 4;16(1):596–615. doi: 10.1016/j.apsb.2025.10.048 (PMC12828083; doi:10.1016/j.apsb.2025.10.048)
Supplement: Multimedia component 1 [file mmc1.docx]

**Supporting Information for**

**Original article**

**Inhalable songorine-integrated lipid nanomedicine for targeted ARDS therapy *via*** **repairing endothelial barrier and inactivating NLRP3 inflammasome**

**Haiyan Wang^a,b†^, Zhi-Chao Sun^a,b†^, Chunlei Dai^c,e†^, Ran Liao^a,b^, Ran Lin^a^, Liying Wang^c^, Wenjun Fu^a^, Ruhe Zhang^c^, Danwen Zheng^a*^, Zhongde Zhang^a,b*^, Jun Wu^c,d*^, Yuntao Liu^a,b*^**

*^a^State Key Laboratory of Traditional Chinese Medicine Syndrome, Guangzhou University of Chinese Medicine, Guangzhou 510006, China*

*^b^Chinese Medicine Guangdong Laboratory, Guangzhou University of Chinese Medicine, Zhuhai 519031, China*

*^c^Bioscience and Biomedical Engineering Thrust, Systems Hub, The Hong Kong University of Science and Technology (Guangzhou), Guangzhou 511400, China*

*^d^Division of Life Science, The Hong Kong University of Science and Technology, Hongkong SAR, 999077, China*

*^e^Department of Hematologic Oncology, Sun Yat-sen University Cancer Center, State Key Laboratory of Oncology in South China, Collaborative Innovation Center for Cancer Medicine, Guangdong Provincial Clinical Research Center for Cancer, Guangzhou 510060, China*

†These authors made equal contributions to this work.

*Corresponding authors.

Tel./fax: +86 13631457177 (Danwen Zheng); +86 020 81887233 (Zhongde Zhang); +86 18718844118 (Jun Wu); +86 13560021023 (Yuntao Liu).

E-mail addresses: [zhengdanwen@gzucm.edu.cn](mailto:zhengdanwen@gzucm.edu.cn) (Danwen Zheng), [doctorzzd99@gzucm.edu.cn](mailto:doctorzzd99@gzucm.edu.cn) (Zhongde Zhang), [junwuhkust@ust.hk](mailto:junwuhkust@ust.hk) (Jun Wu), and [liuyuntao@gzucm.edu.cn](mailto:liuyuntao@gzucm.edu.cn) (Yuntao Liu).

Runnng title：Inhalable songorine-integrated lipid nanomedicine facilitates targeted ARDS therapy

Table S1 The primers used in this study for RT-qPCR assays.

| Primer name | Sequence (5’-3’) |
| --- | --- |
| M-*Tnfa*-F | TTGTCTACTCCCAGGTTCTCT |
| M-*Tnfa*-R | GAGGTTGACTTTCTCCTGGTATG |
| M-*Il1b*-F | GGTGTGTGACGTTCCCATTA |
| M-*Il1b*-R | ATTGAGGTGGAGAGCTTTCAG |
| M-*Tubulin*-F | AGCAGCTACTTTGTGGAGTG |
| M-*Tubulin*-R | TCGGAGATGCGCTTGAATAG |
| M-*Nos2*-F | GGAATCTTGGAGCGAGTTGT |
| M-*Nos2*-R | CCTCTTGTCTTTGACCCAGTAG |
| M-*Il6*-F | CTTCCATCCAGTTGCCTTCT |
| M-*Il6*-R | CTCCGACTTGTGAAGTGGTATAG |
| M-*Tjp1*-F | ACTCCCACTTCCCCAAAAAC |
| M-*Tjp1*-R | CCACAGCTGAAGGACTCACA |
| M-*Cdh5*-F | GCCACTGTCTTGTACCAAATCGT |
| M-*Cdh5*-R | AGCCTGTTTCTCTCGGTCCA |
| M-*Cldn5*-F | ACTGCCTTCCTGGACCACAAC |
| M-*Cldn5*-R | CGCCAGCACAGATTCATACACCT |
| H-*TJP1*-F | CAACATACAGTGACGCTTCACA |
| H-*TJP1*-R | CACTATTGACGTTTCCCCACTC |
| H-*CDH5*-F | AAGCGTGAGTCGCAAGAATG |
| H-*CDH5*-R | TCTCCAGGTTTTCGCCAGTG |
| H-*CLDN5*-F | CTCTGCTGGTTCGCCAACAT |
| H-*CLDN5*-R | CAGCTCGTACTTCTGCGACA |
| H-*ACTIN*-F | CCTGGCACCCAGCACAAT |
| H-*ACTIN*-R | GGGCCGGACTCGTCATAC |
| H-*BCL2*-F | AGTACCTGAACCGGCATCTG |
| H-*BCL2*-R | CATGCTGGGGCCATATAGTT |
| H-*BAX*-F | CATGAAGACAGGGGCCCTTT |
|  |  |
| Table S1 (continued) | |
| Primer name | Sequence (5’-3’) |
| H-*BAX*-R | AAACACAGTCCAAGGCAGCT |
| M-*Bcl2*-F | GTCGCTACCGTCGTGACTTC |
| M-*Bcl2*-R | CAGACATGCACCTACCCAGC |
| M-*Bax*-F | ATGCTTAGGGTAGCGGAGC |
| M-*Bax*-R | TGCGGATTGCCTGAGTGTC |
| M-*Nlrp3*-F | CCAGAAACTGTGACTGTACATTGG |
| M-*Nlrp3*-R | TTGGTCCCACACAAGCCTTT |
| M-*Casp1*-F | GAAACGCCATGGCTGACAAG |
| M-*Casp1*-R | ACTTGAGGGTCCCAGTCAGT |

Table S2 Encapsulation efficiency and drug-loading capacity of Son-lipo

| Drug-to-lipid ratio | Drug (mg) | Drug loading content (mg) | Encapsulation efficiency (%) | Drug loading efficiency (%) |
| --- | --- | --- | --- | --- |
| 1:13 | 10 | 1.11 | 11.1 | 0.8 |
| 1:8 | 10 | 6.48 | 64.8 | 7.4 |
| 1:5 | 10 | 7.75 | 77.5 | 12.5 |


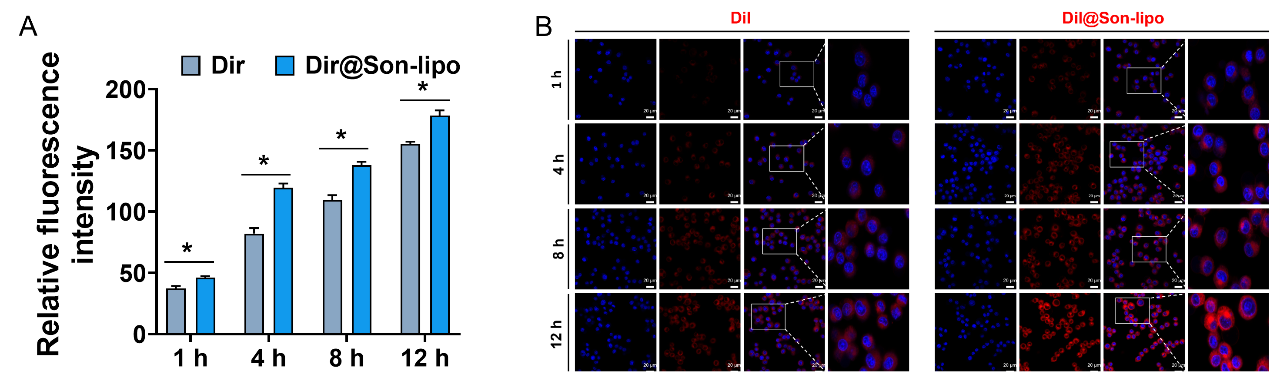


Figure S1 Uptake of Son-lipo by cells. (A) The statistical analysis of cellular uptake in Figure 1F (*n*=5). (B) Uptake of Son-lipo by MH-S cells. Images were captured using a 40× oil lens of a confocal microscope. Scale bar=20 μm. Data are represented as mean ± SD. **P* < 0.05.


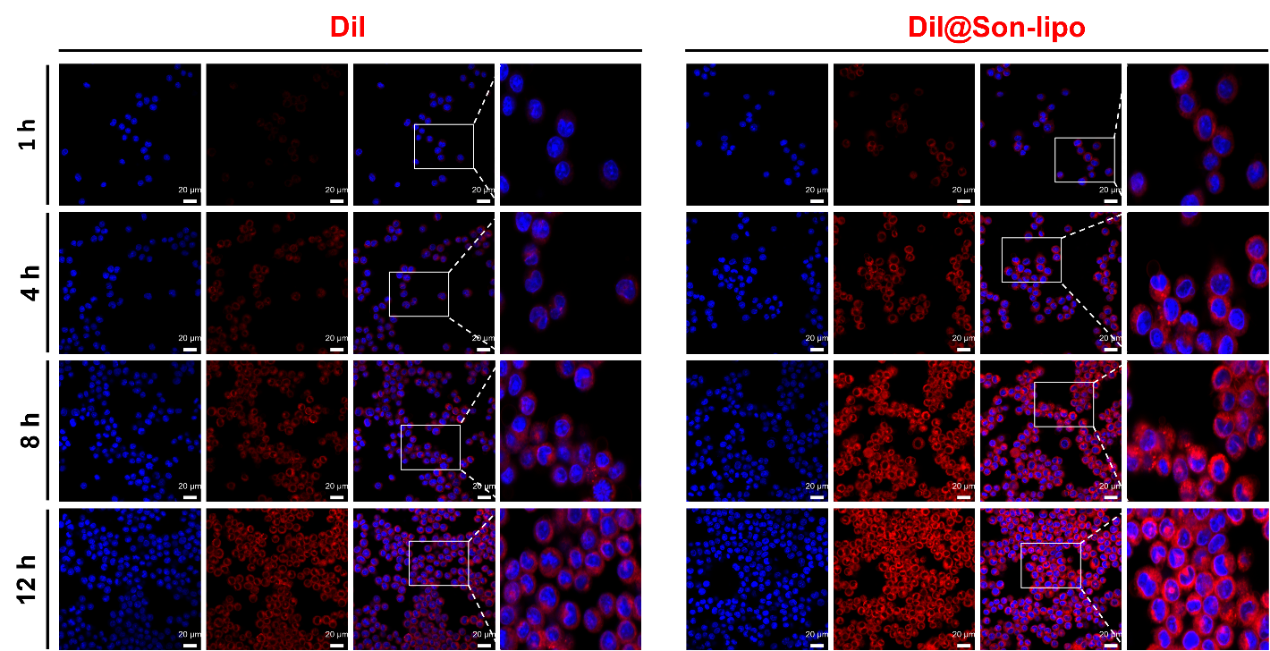


Figure S2 Uptake of Son-lipo by RAW264.7 cells. Images were captured using a 40× oil lens of a confocal microscope. Scale bar=20 μm.


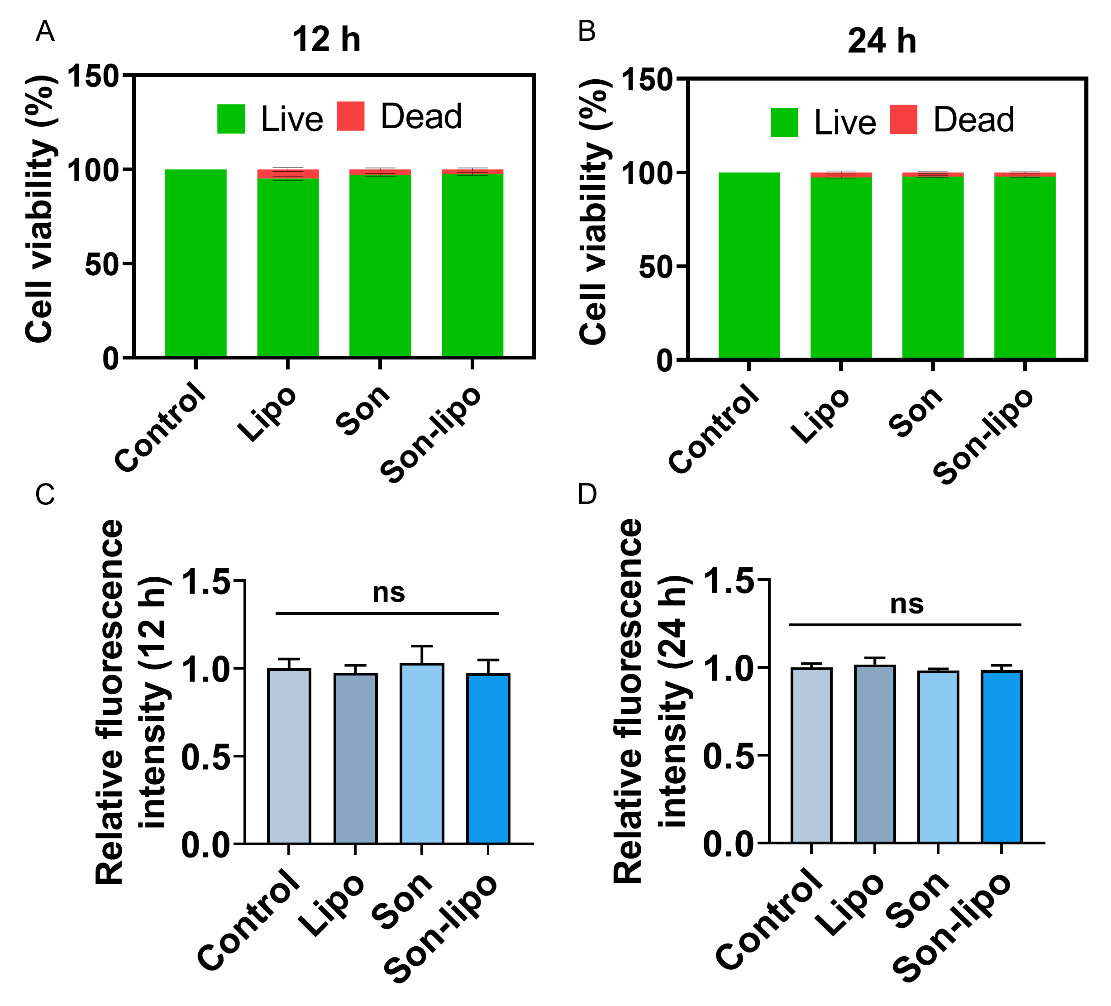


Figure S3 The statistical analysis of (A‒B) live/dead staining in Figure 2C, (C‒D) cytoskeleton staining in Figure 2D (*n*=5). Data are represented as mean ± SD. ns, not significant.


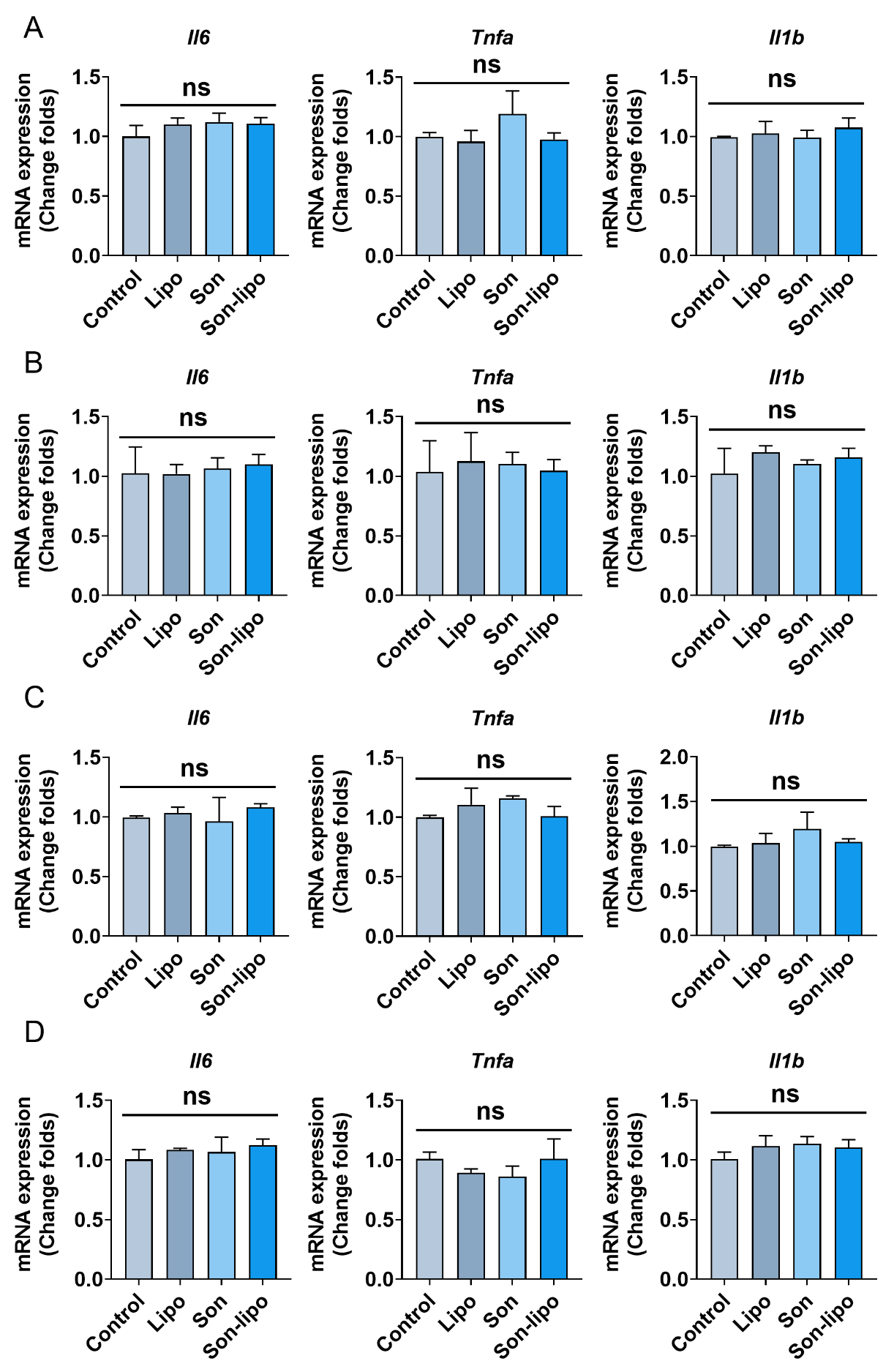


Figure S4 Biosafety evaluation of drugs in mice. (A‒D) The RT-qPCR analysis of inflammatory factors, *Il6*, *Tnfa*, and *Il1b*, in heart, liver, spleen, and kidney, respectively (*n*=3). Internal control, *α-Tubulin*. Data are represented as mean ± SD. ns, not significant.


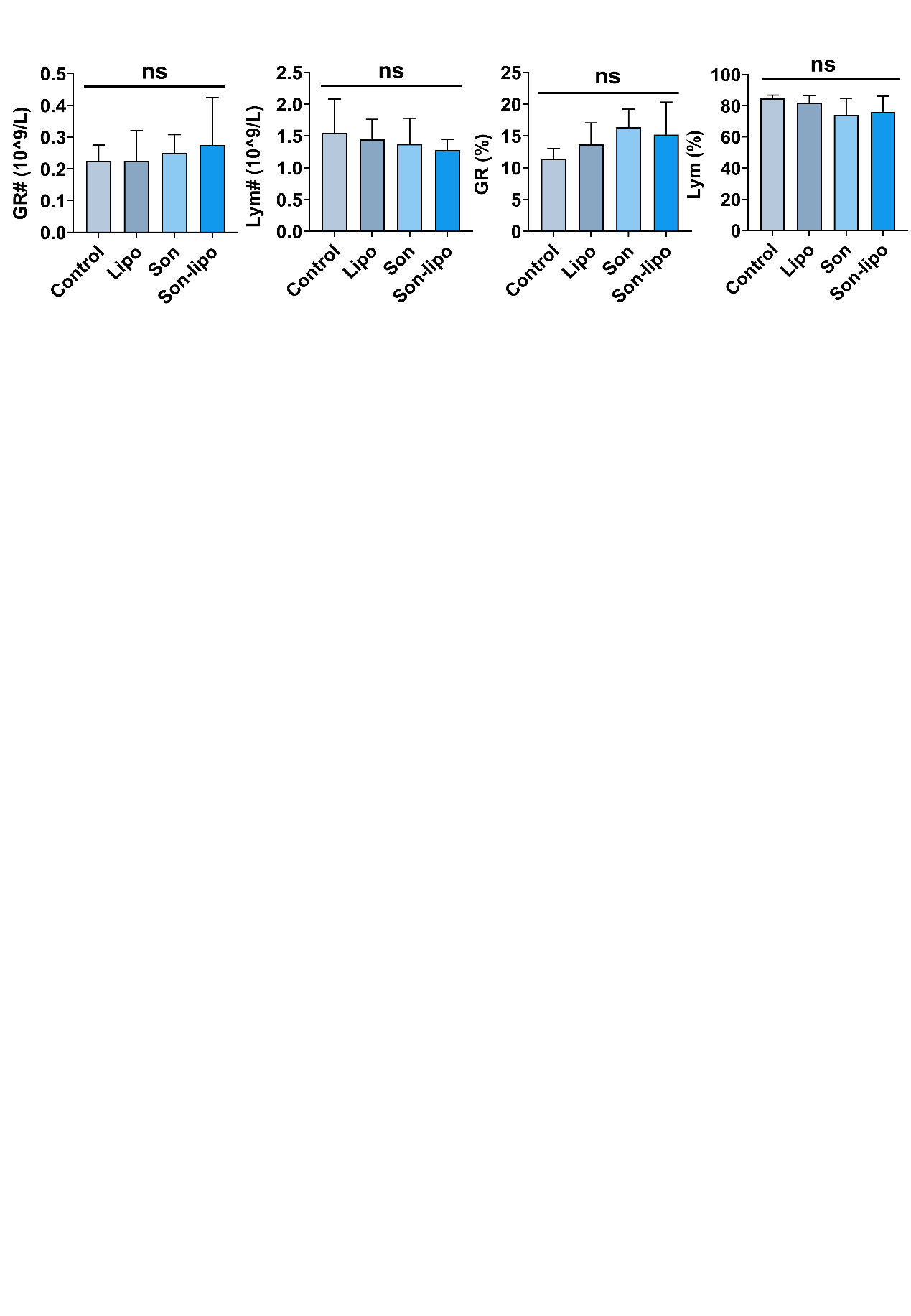


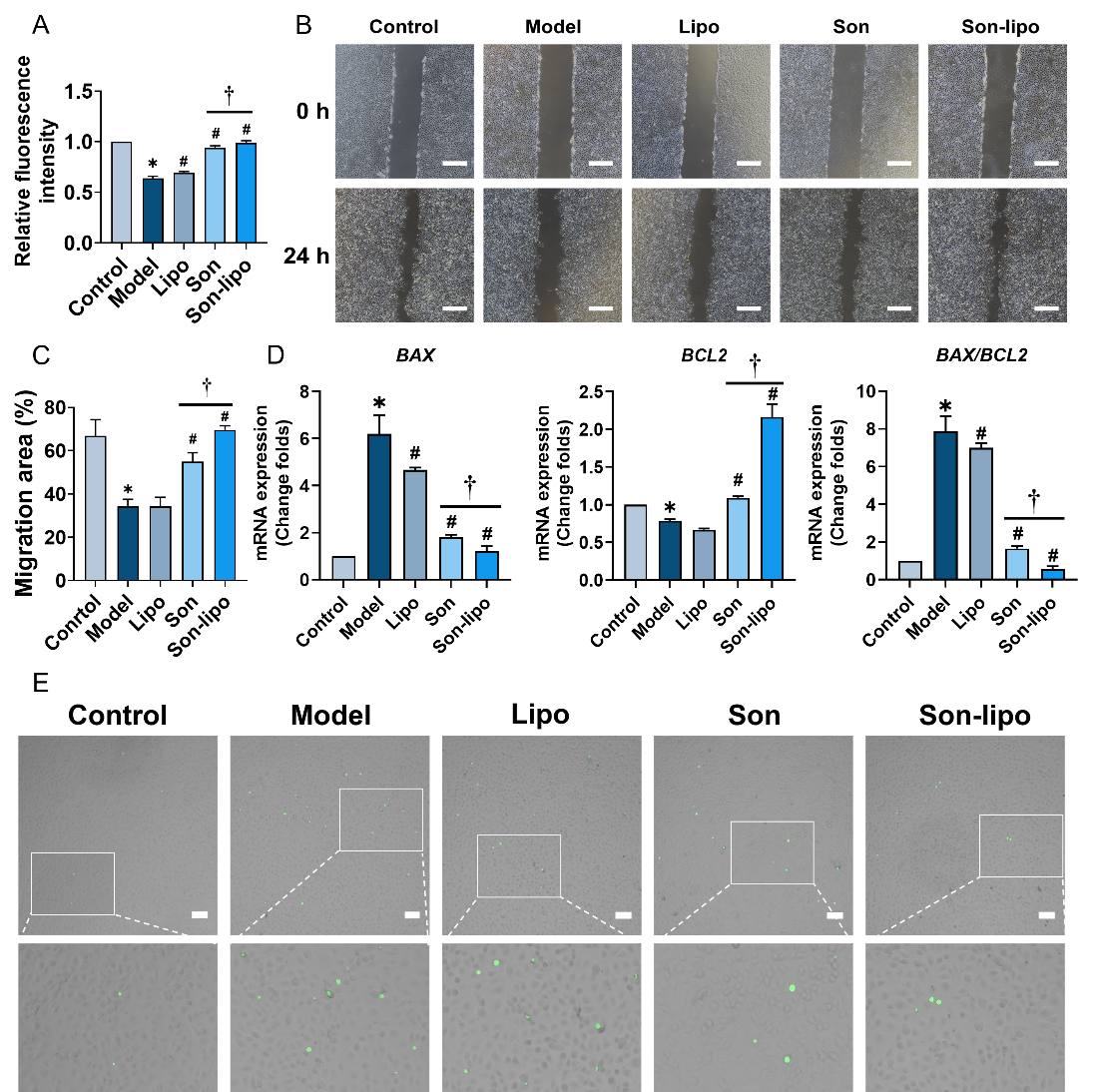
Figure S5 Inflammatory markers in common hematological examination, including neutrophil count (GR#), lymphocyte count (Lym#), neutrophil percentage (GR%), and lymphocyte percentage (Lym%) (*n*=4). ns, not significant.

Figure S6 (A) The statistic of immunofluorescent staining of tight junction factor ZO-1 in Figure 4A (*n*=5). (B‒C) Results of the cell migration test (*n*=5‒6). Scale bar=500 μm. (D) The RT-qPCR analysis of apoptosis factors, *BAX*, *BCL2*, and *BAX/BCL2*, in HUVEC cells, respectively (*n*=3). Internal control, *β-ACTIN*. (E) TUNEL staining of an endothelial cell. Scale bar=100 μm. Data are represented as mean ± SD. **P* < 0.05 *vs.* Control*.* #*P* < 0.05 *vs.* Model. †*P < 0.05*.

**
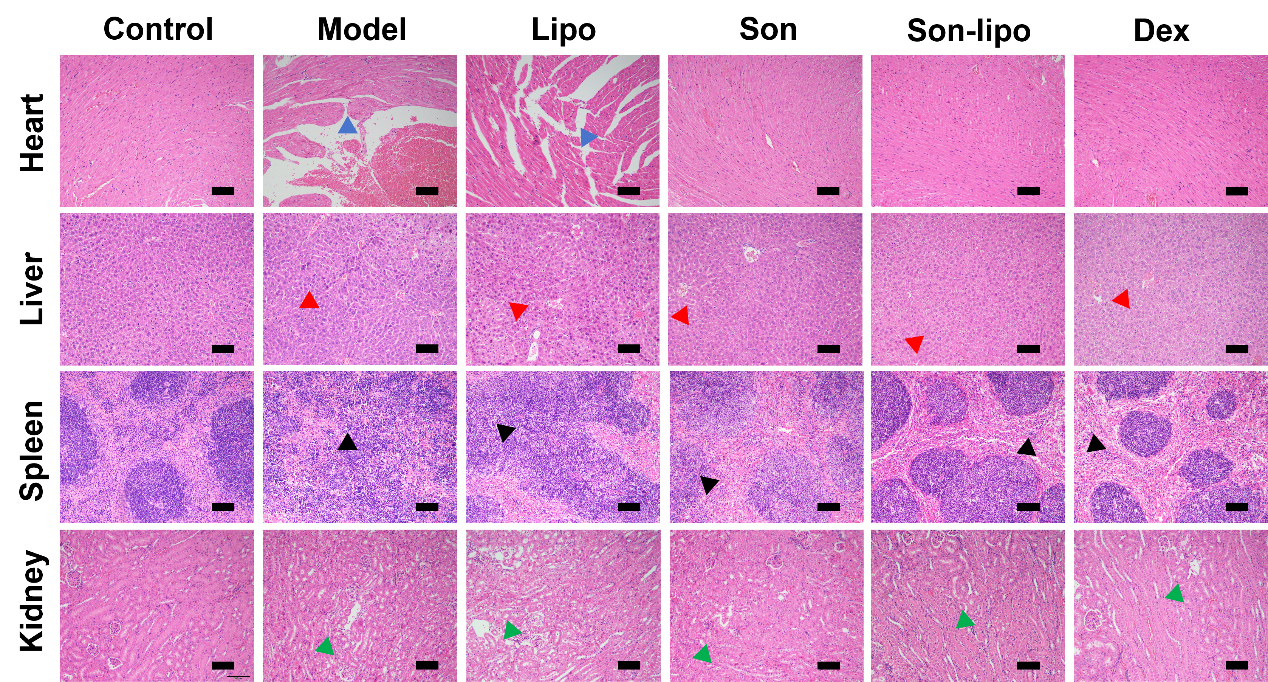
**

Figure S7 Son-lipo attenuated pathological injuries in the heart, liver, spleen, and kidneys of sepsis mice. HE staining of heart, liver, spleen, and kidney tissues. Blue arrows, myocardial fiber disruption in the heart. Red arrows, hepatocyte swelling in the liver. Black arrows, inflammatory cell proliferation in the spleen. Green arrows, tubular epithelial vacuolation and interstitial edema in the kidney. Scale bar=100 μm.

**
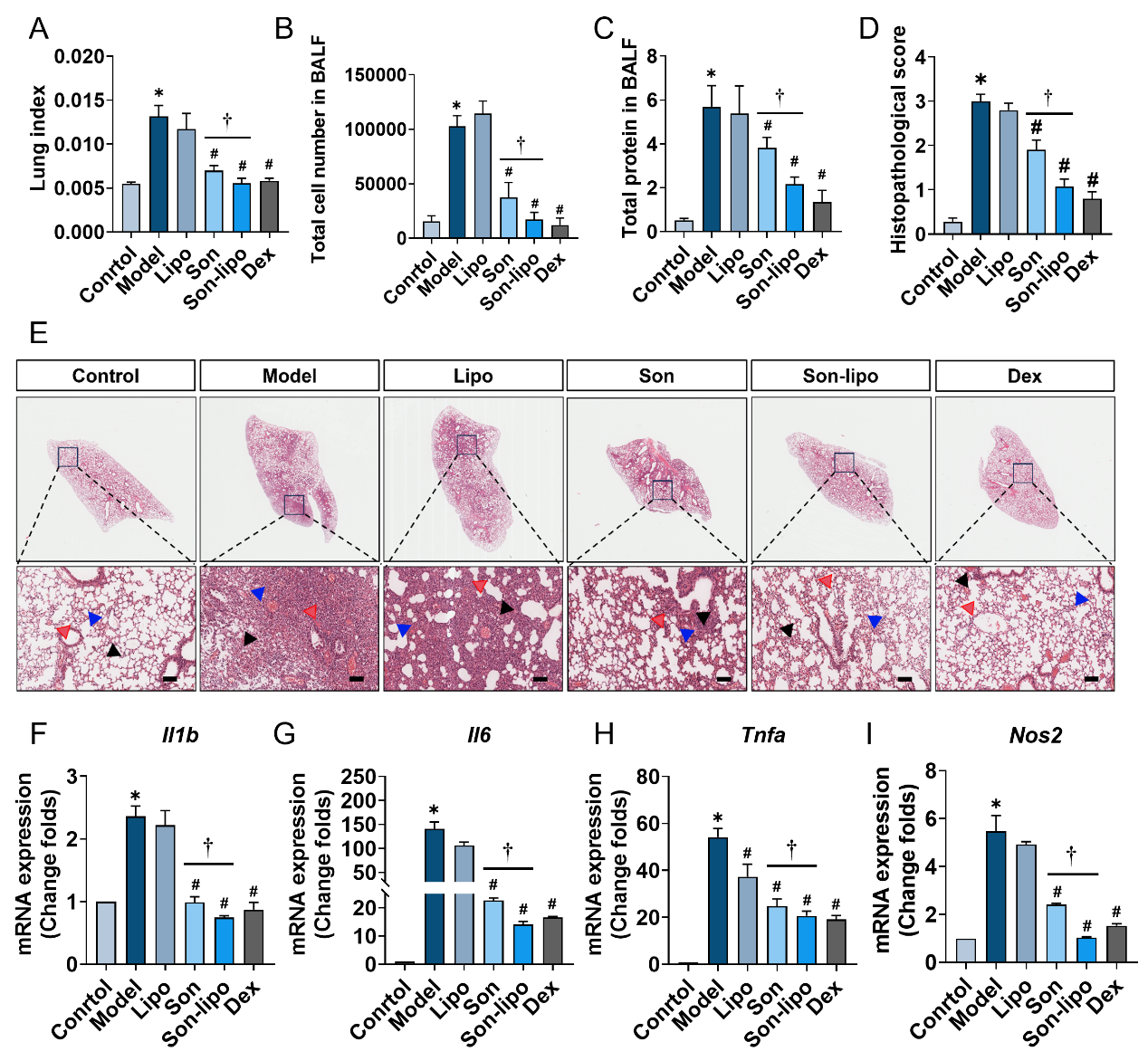
**

Figure S8 Son-lipo ameliorated the phenotypes of LPS-induced ARDS *in vivo*. (A) Lung index of mice (*n*=5‒6). (B) Total cell number and (C) protein content in BALF (*n*=5‒6). (D‒E) HE staining and pathological scores of lung tissues. Black arrows, the degree of inflammatory cell infiltration and destroyed alveoli structures. Red arrows, alveoli wall. Blue arrows, the degree of alveoli leakage. Scale bar=1000 μm for the upper panels and 100 μm for the lower panels (*n*=5). (F‒I) The change in folds of mRNA levels of inflammatory factors, *Il1b*, *Il6, Tnfa*, and *Nos2*, in lung tissues (*n*=4). Data are represented as mean ± SD. **P* < 0.05 *vs.* Control. #*P* < 0.05 *vs.* Model. †*P* <0.05.


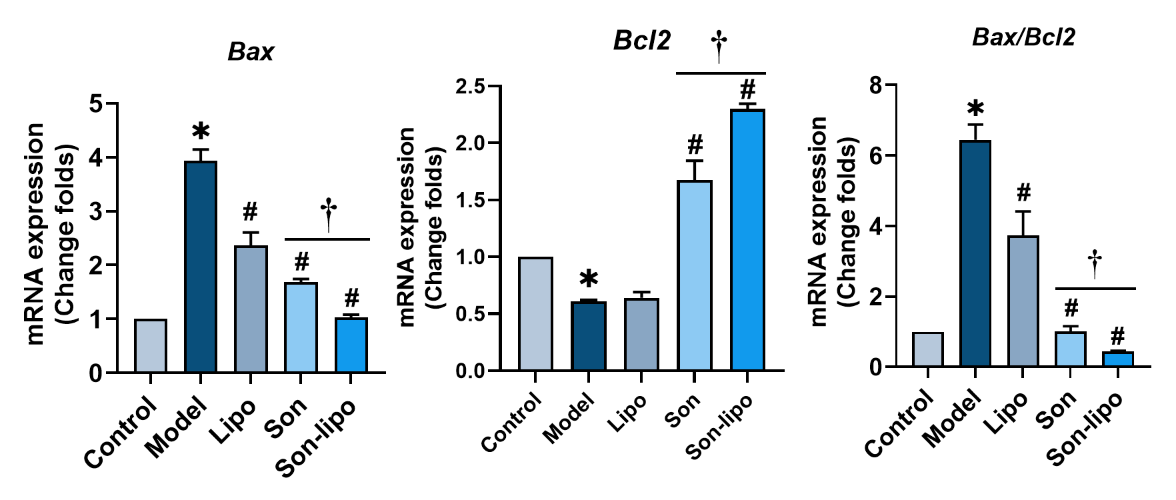


Figure S9 The RT-qPCR analysis of apoptosis factors, *Bax*, *Bcl2* and *Bax/Bcl2*, in lung tissue (*n*=3). Internal control, *α-Tubulin*. Data are represented as mean ± SD. **P* < 0.05 *vs.* Control*. #P* < 0.05 *vs.* Model*.* †*P* < 0.05.


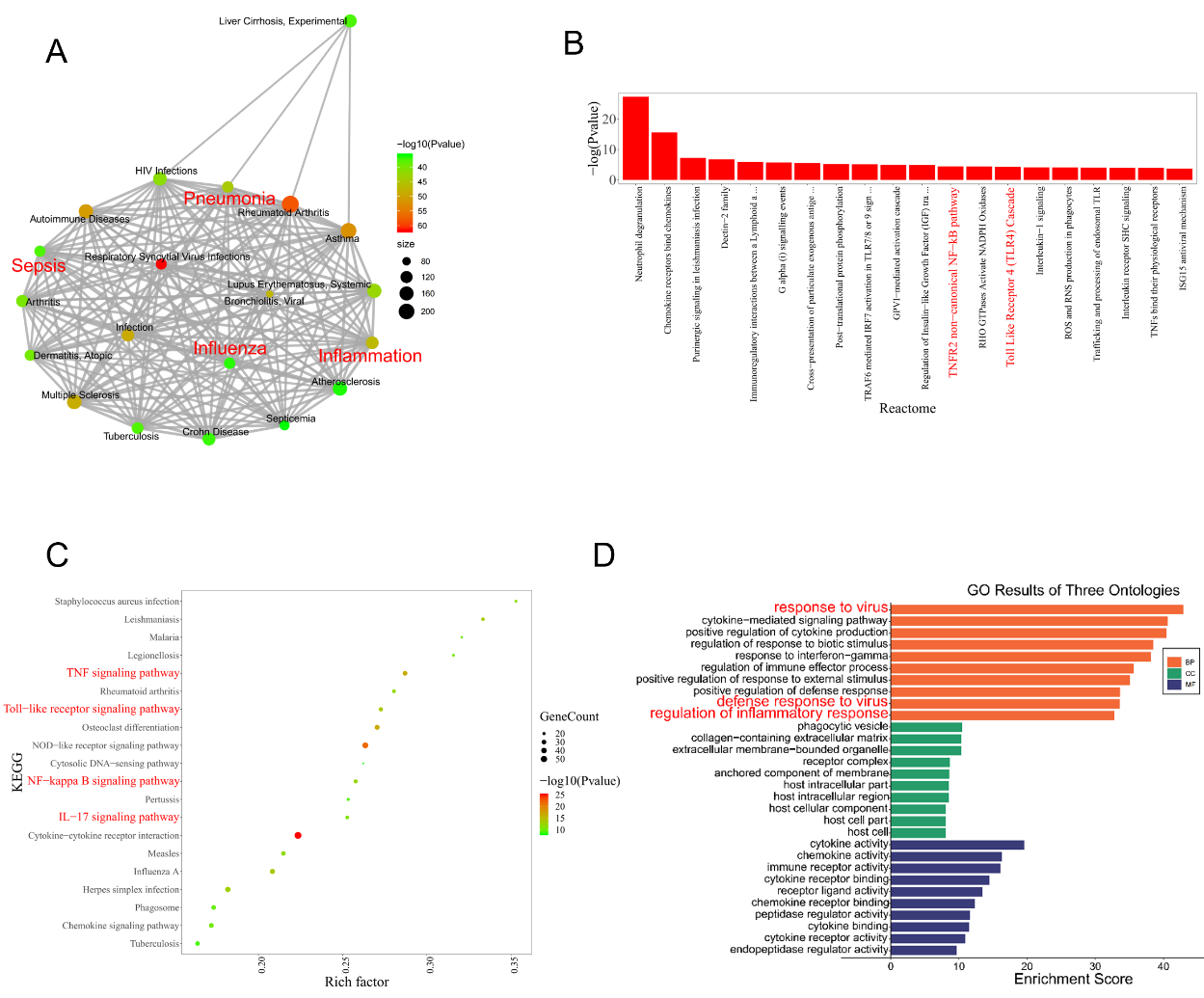


Figure S10 Analysis of 1293 DEGs after taking the intersection of the venn diagrams in Figure 7. The upregulated DEGs in the Model were subjected to disease enrichment analysis in a net plot (A), reactome analysis in a bubble diagram (B), KEGG analysis in a bubble diagram (C), and GO analysis in a bar plot (D). Main inflammatory terms were marked in red.


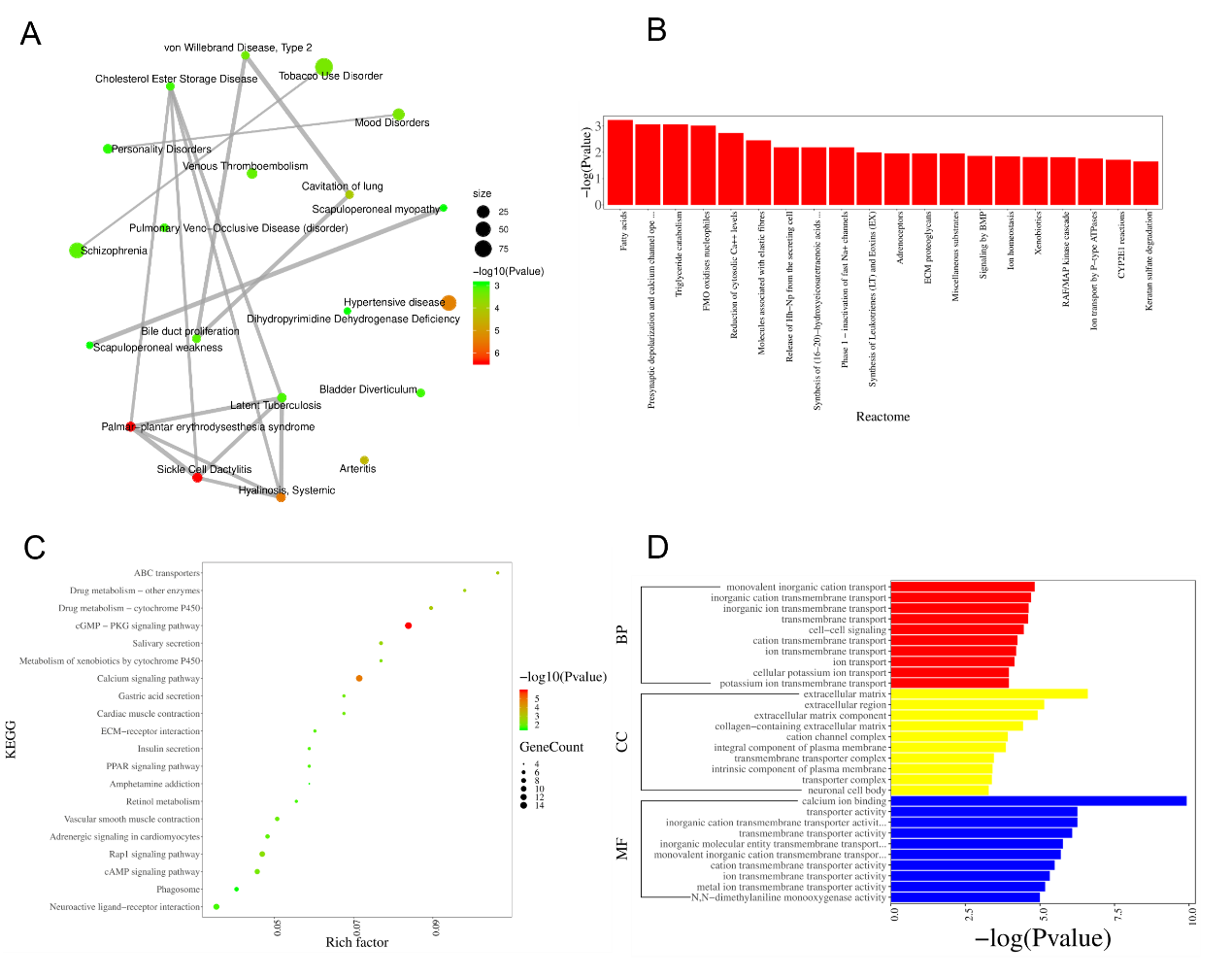


Figure S11 Analysis of 1293 DEGs after taking the intersection of the venn diagrams in Figure 7. The downregulated DEGs in the Model were subjected to disease enrichment analysis in a net plot (A), reactome analysis in a bubble diagram (B), KEGG analysis in a bubble diagram (C), and GO analysis in a bar plot (D). Main inflammatory terms were marked in red.


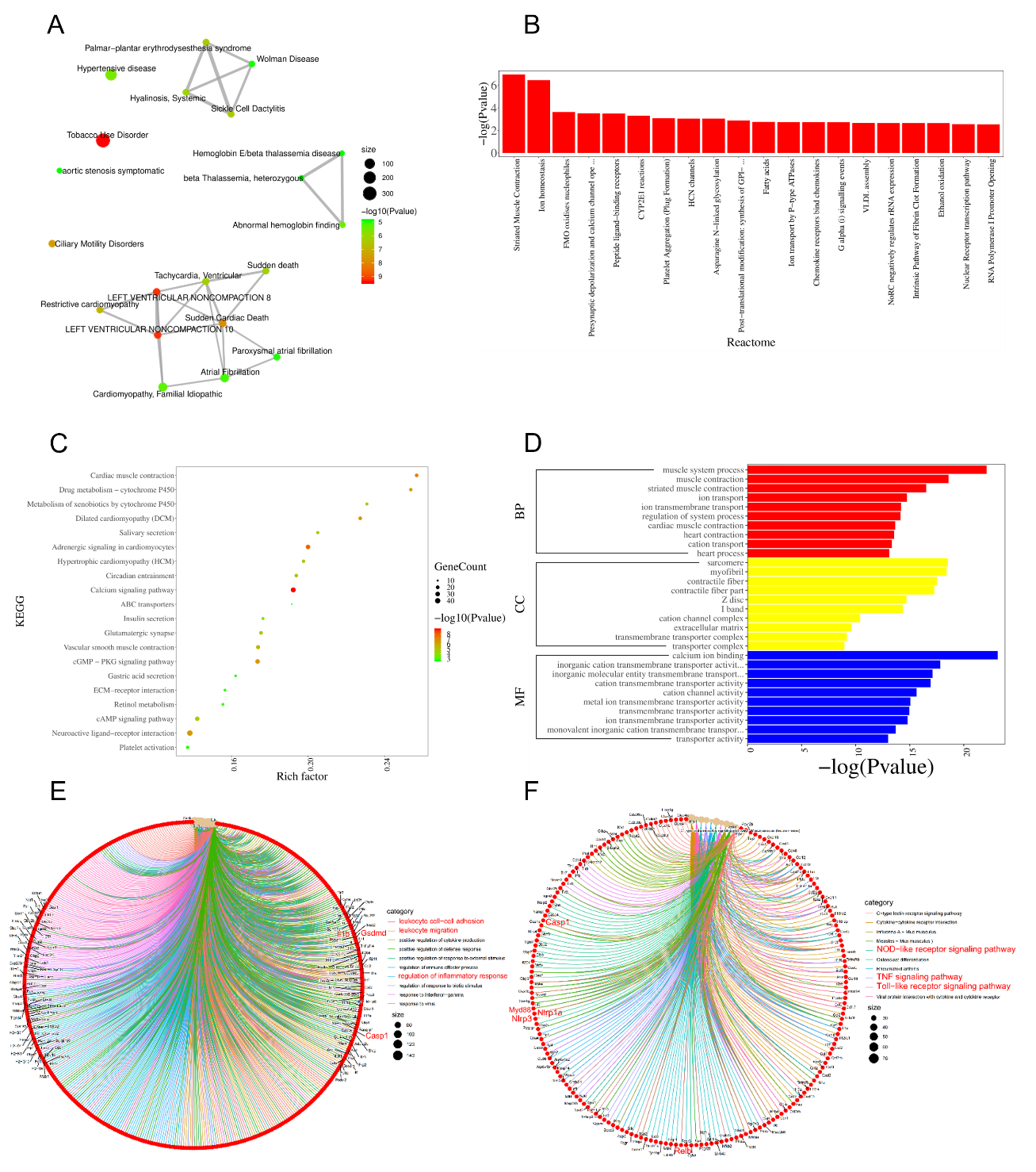


Figure S12 Analysis of DEGs between Son-lipo and Model. The downregulated DEGs in the Model subjected to disease enrichment analysis in a net plot (A), reactome analysis in a bubble diagram (B), KEGG analysis in a bubble diagram (C), and GO analysis in a bar plot (D). The upregulated DEGs in the Model were subjected to GO-BP (E) and KEGG (F) analysis in the rainbow plot. Main inflammatory terms were marked in red.


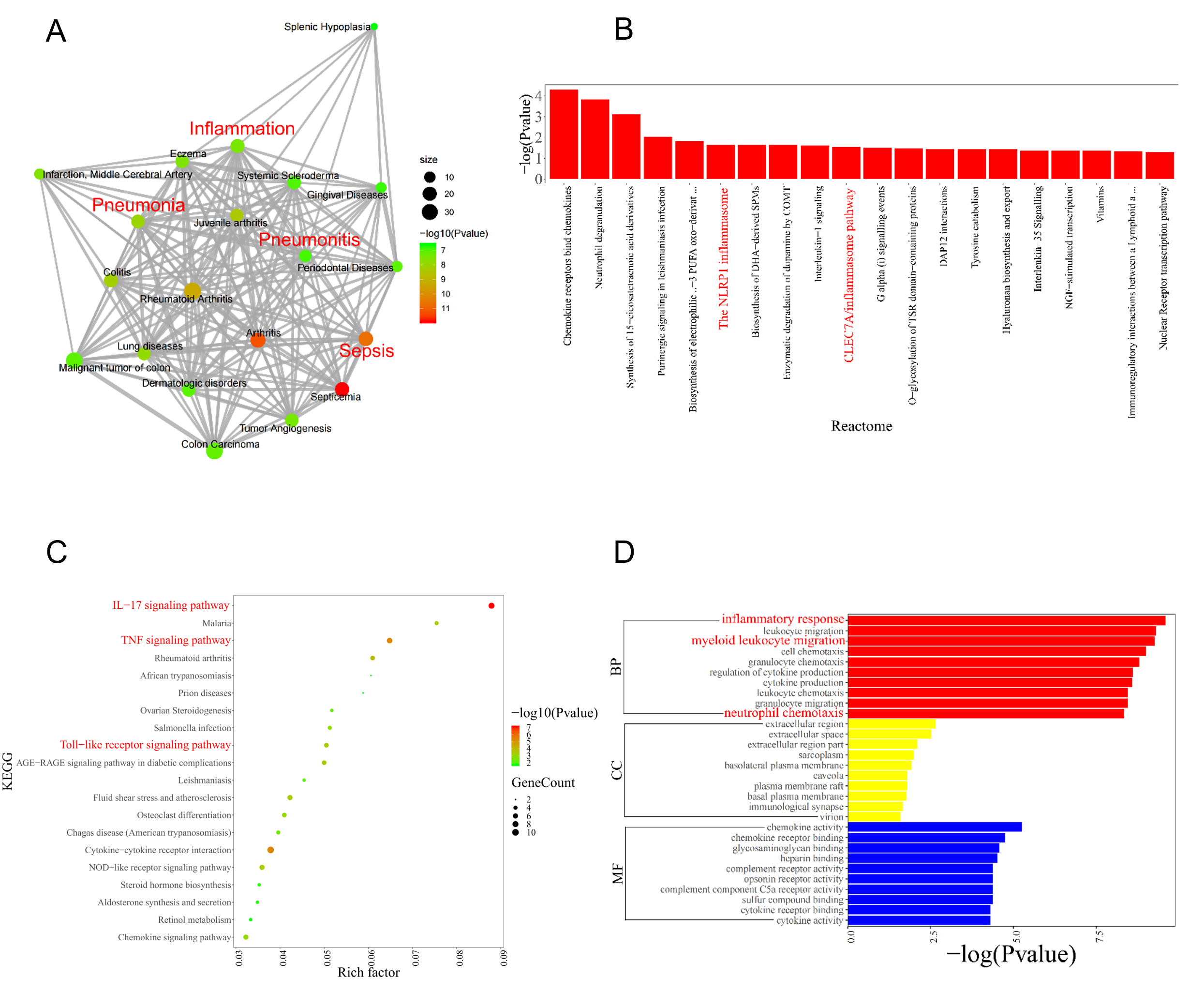


Figure S13 Analysis of DEGs between Son-lipo and Son. The downregulated DEGs in the Son-lipo subjected to disease enrichment analysis in a net plot (A), reactome analysis in a bubble diagram (B), KEGG analysis in a bubble diagram (C), and GO analysis in a bar plot (D). Main inflammatory terms were marked in red.


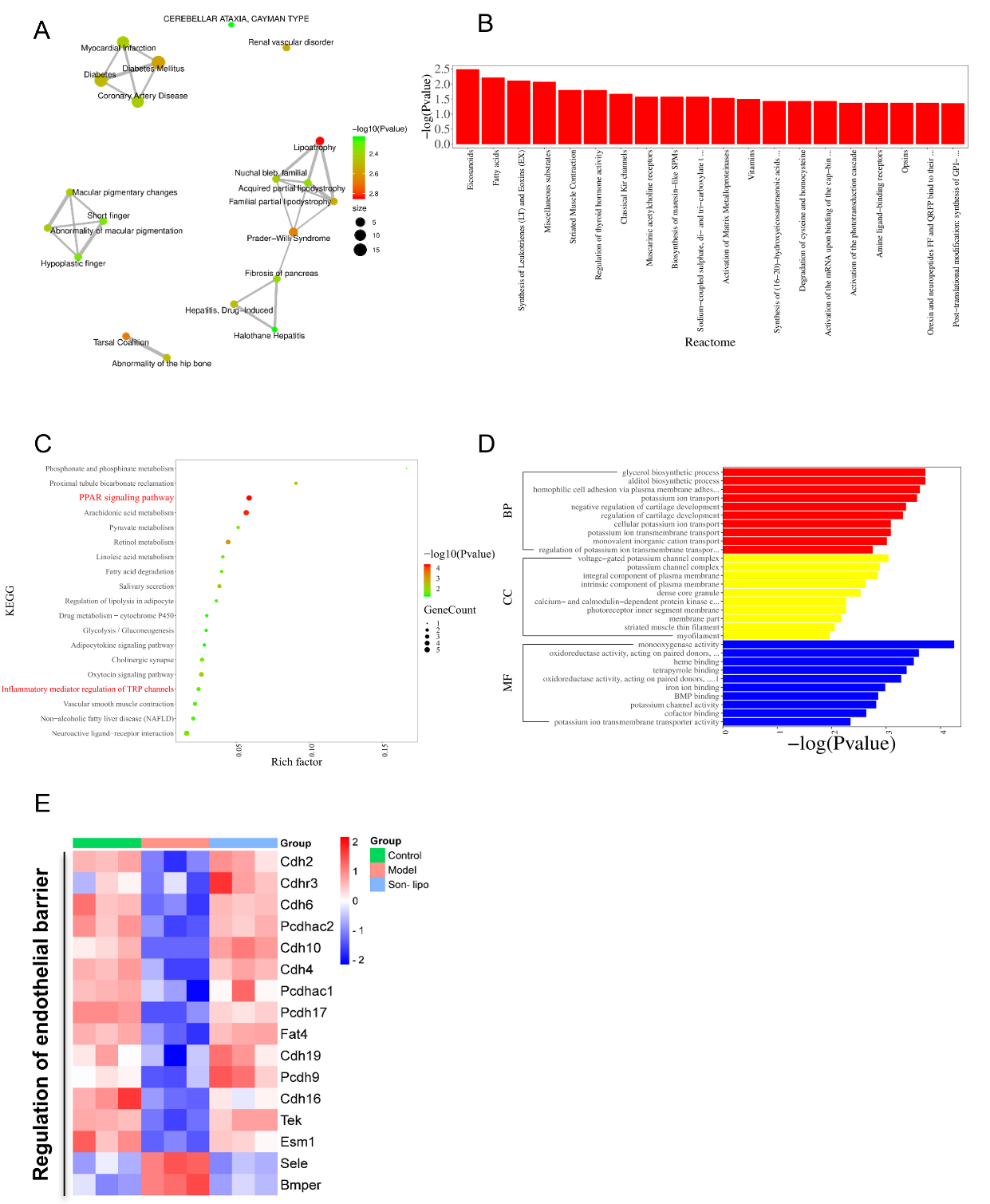


Figure S14 Analysis of DEGs in Son-lipo and Son. The upregulated DEGs in the Son-lipo subjected to disease enrichment analysis in a net plot (A), reactome analysis also in a bubble diagram (B), KEGG analysis in a bubble diagram (C), and GO analysis in a bar plot (D). Main inflammatory terms were marked in red. (E) Cluster analysis of vascular endothelial barrier-associated DEGs among Control, Model, and Son-lipo.

**Reference**

1. Liu PY, Shen J, Cao JK, Jiang WB. p-Coumaric acid-loaded nanoliposomes: Optimization, characterization, antimicrobial properties and preservation effects on fresh pod pepper fruit. *Food Chem.* 2024, **435**:137672.
